# Mixed-methods research to support the use of new lymphoma-specific patient-reported symptom measures derived from the EORTC item library

**DOI:** 10.1186/s41687-024-00683-2

**Published:** 2024-01-22

**Authors:** Jessica T. Markowitz, Flora Mazerolle, Teya Lovell, Lisa M. Hess, Paolo B. Abada, Antoine Regnault, Nalin Payakachat

**Affiliations:** 1 Modus Outcomes, A Division of THREAD Research, Cambridge, MA USA; 2 Modus Outcomes, A Division of THREAD Research, Lyon, France; 3 Eli Lilly and Company, Indianapolis, IN USA; 4 Loxo@Lilly, Indianapolis, IN USA

**Keywords:** Patient-reported outcomes, Chronic lymphocytic leukemia, Small lymphocytic lymphoma, Mantle cell lymphoma

## Abstract

**Background:**

No specific measures exist to assess patient-reported symptoms experienced by individuals with chronic lymphocytic leukemia (CLL)/small lymphocytic lymphoma (SLL) or mantle cell lymphoma (MCL). This study was conducted to elicit patient-reported CLL/SLL- and MCL-related symptoms and their impact on patients’ lives. The study qualitatively and quantitatively evaluated sets of conceptually-selected EORTC Item Library items for assessing CLL/SLL- and MCL-related symptoms.

**Methods:**

The qualitative component of the research included a literature review, clinician consultations, and patient interviews. Concepts important to patients were identified and coded; cognitive debriefing of the selected library items was completed with patients. CLL/SLL and MCL-related symptoms and impacts were organized in a structured conceptual model, which was mapped to item sets from the Item Library. The quantitative component comprised exploratory macro-level Rasch measurement theory (RMT) analysis conducted to provide supportive quantitative insight on the item sets.

**Results:**

41 patients (21-MCL; 20-CLL/SLL) and 5 clinicians participated in the qualitative study; 57 unique patients (30-MCL; 27-CLL/SLL) completed the EORTC items. The conceptual models generated from the qualitative work included symptoms and functional impacts of CLL/SLL and MCL. Symptom domains included swollen lymph nodes, B symptoms, abdominal issues, pain, fatigue, subjective cognitive impairment, anemia-related symptoms, bleeding, infection, and other issues (appetite loss, temperature fluctuation, rash, weight gain, sleep problems, cough). Impacts included physical function, role function, and other functions (psychological, social). Cognitive debriefing demonstrated that the separate item sets for CLL/SLL and MCL-related symptoms were well understood and aligned with patients’ experiences. All selected items were included in the conceptual models. The exploratory RMT analysis showed that the item sets provided adequate coverage of the continuum of CLL/SLL- and MCL-related symptom severity.

**Conclusions:**

This study gathered qualitative and early quantitative evidence supporting use of the EORTC Item Library to assess CLL/SLL- and MCL-related symptoms and impacts. These items are promising candidates for measurement of patient-reported disease symptoms in these populations. A larger sample size will be essential to establish the psychometric properties necessary to support use in clinical trials.

**Plain English summary:**

Patients who suffer from rare cancers of the blood, bone marrow, and lymph nodes can experience chronic and debilitating symptoms. At present, however, there are no dedicated instruments for assessing the patient’s experience of symptoms of conditions like chronic lymphocytic leukemia (CLL)/small lymphocytic lymphoma (SLL) or mantle cell lymphoma (MCL), or for assessing their impact on patients’ lives. This research project aimed to address that need. The researchers selected relevant and clinically meaningful symptoms from the EORTC Item Library that assess fatigue, B symptoms, and CLL/SLL- and MCL-specific symptoms. Using patients and clinician interviews as well as quantitative analyses, the research revealed no major concerns with using these item sets to assess symptoms of CLL/SLL and MCL. Interviews with patients demonstrated that the separate item sets for CLL/SLL and MCL-related symptoms were well understood and aligned with patients’ experiences. All selected items were included in the conceptual models. Item sets identified in this study can potentially be used to assess patient-reported symptom endpoints in clinical trial settings in these disease areas.

**Supplementary Information:**

The online version contains supplementary material available at 10.1186/s41687-024-00683-2.

## Introduction

B-cell malignancies, which include non-Hodgkin lymphomas (NHLs) and chronic lymphocytic leukemia (CLL), are challenging to treat and generally considered incurable [1]. Newer therapies such as Bruton’s tyrosine kinase inhibitors (e.g., covalent BTKis ibrutinib; acalabrutinib; zanubrutinib; and the non-covalent BTKi, pirtobrutinib), have shown improved outcomes in the treatment of diverse types of B-cell malignancies, including CLL, small lymphocytic lymphoma (SLL), and mantle cell lymphoma (MCL) [2–5].

Evaluation of new therapies in cancer clinical trials increasingly includes the evaluation of the benefit in terms of patient-reported outcomes (PRO). The United States Food and Drug Administration has identified core PRO concepts including disease-related symptoms, symptomatic adverse events, overall side effect impact, physical function, and role function for use in clinical trials to address heterogeneity in PRO assessment strategies that lessens the regulatory utility of PRO data from cancer trials [6]. Robust demonstration of benefits in these PRO concepts requires rigorous measurement strategy, involving clear PRO hypotheses of treatment benefit underpinned by definitions of the precise concepts of study, and selection of fit-for-purpose measures for these concepts [7, 8].

Evaluating benefits in clinical trials in hematologic malignancies such as CLL/SLL and MCL with PRO measures requires careful consideration of the key concepts to be assessed, especially for symptoms related to these specific B-cell malignancies, followed by selection of the appropriate measures for this purpose. Little research has been conducted to investigate the experience of patients with CLL/SLL or MCL and limited PRO measures specific to these contexts are available. Some PRO measures are in development for symptoms of B-cell malignancies more broadly (such as the European Organization for Research and Treatment of Cancer (EORTC) Quality of Life questionnaires (QLQ) in CLL (QLQ-CLL17) [9], high grade NHL (QLQ-NHL-HG29) [10] and low grade NHL (QLQ-NHL-LG20) [10]), but they are not yet fully validated. Additionally, they focus on broad concepts such as health-related quality of life rather than key disease symptoms relevant to B-cell malignancies [9].

Item libraries compiling PRO items from existing measures offer a promising solution for assessing a targeted PRO concept in the absence of a readily available PRO measure [11]. Previous research has illustrated how the EORTC Item Library can be used in conjunction with Quality of Life Questionnaire-Core 30 items (QLQ-C30) to construct new symptom and impact assessments in cancer [12–14].

The current study used a mixed-methods, patient-centered research approach to address two key research questions: (1) What are the key PRO concepts important to patients with CLL/SLL or MCL, especially related to their symptoms; and (2) Would selection of items from the EORTC Item Library form a sensible basis for creating a measure of these disease-related symptoms in MCL, CLL and SLL for use in clinical trials.

## Methods

### Study design

**Fig. 1:**
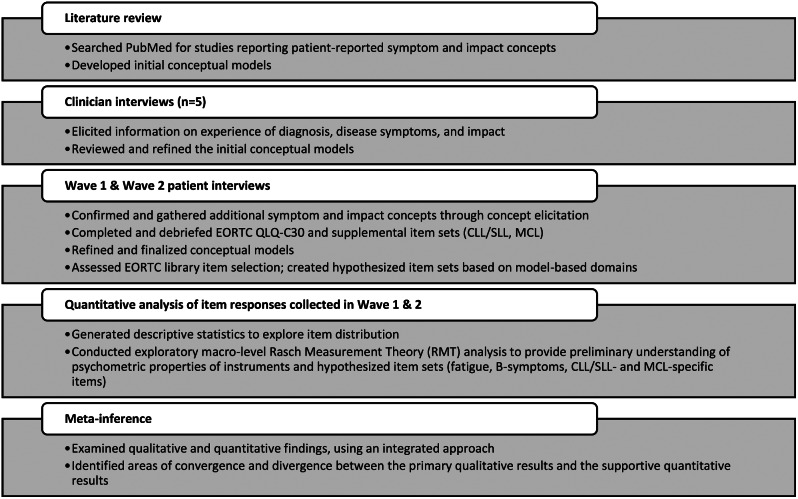
Mixed methods research process to support EORTC Item library selection in CLL/SLL and MCL

This mixed methods study comprised a primary qualitative research strand and a secondary exploratory quantitative (psychometrics) research strand. Decisions regarding the conceptual models and item sets were made primarily based on qualitative findings, with quantitative findings playing a supplementary role. An overview of the research process is outlined in Fig. 1.

### Mixed methods: primary qualitative strand

#### Literature review

A targeted literature search was conducted in June 2020 to gather patient-derived concepts to refine the measurement strategy and develop draft conceptual models of disease symptom and impact experience (see supplemental materials S-0^1^). Articles were screened by title and abstract; full texts of included articles were subsequently reviewed. Concepts specific to disease symptoms and impacts were extracted.

#### Clinician interviews

Five clinicians board-certified in hematology and/or medical oncology with a primary practice in hematology and experience treating patients with CLL/SLL or MCL in the past year in the United States were interviewed in December 2020 – January 2021. Clinicians provided informed consent for the interviews, which were 60-minutes long and conducted online, using a semi-structured interview guide. Interviewers took detailed notes, which were compiled in a summary document. Interviews sought to gather additional data around symptoms, impacts, and treatment benefit/risks of CLL/SLL and MCL. Clinicians also reviewed and provided feedback on the concepts identified from the literature and the preliminary conceptual models, which were refined based on this expert feedback and patient interviews.

#### Patient interviews

An observational, descriptive, cross-sectional study was conducted with people living with CLL/SLL or MCL. Wave 1 of the study (*n* = 20 CLL/SLL, *n* = 21 MCL) sought to identify and confirm relevant symptom and impact concepts, refine conceptual models, and determine the relevance and acceptability of a preselected set of items from the EORTC Item Library. The EORTC Item Library comprises over 850 unique items in 110 languages, which can be used to supplement the core QLQ-C30 questionnaire [15]. Separate relevant symptom item sets in CLL/SLL and MCL were preselected as a starting point for the research, based on initial clinical hypotheses regarding the experience of patients living with these conditions (see supplemental materials S-0^3^).

Wave 2 (*n* = 11 CLL/SLL, *n* = 12 MCL) was originally planned to include further debriefing and item completion if new items were added based on Wave 1 findings, and item completion only to gather data for quantitative analysis if no new items were added after Wave 1.

People with active CLL/SLL or MCL living in the United States were recruited from January – October 2021 using resources such as recruiter databases, patient associations, clinician referrals, and social media. Eligible participants were aged ≥ 18 years of age or older; spoke, read, and understood English; had a clinically confirmed diagnosis of CLL/SLL or MCL; were willing to participate in one or two interviews; and provided written informed consent prior to participation. After recruitment had begun, the researchers expanded the eligibility criteria in a protocol amendment to include patients who were in remission anywhere from one month to 12 months to address recruitment challenges. Participants were excluded if their disease was currently in remission or if they had been diagnosed with a second primary cancer within the last 5 years (see supplemental materials S-0^2^).

Open-ended, semi-structured interviews were conducted online, recorded, and transcribed verbatim. Concept elicitation sought to identify symptoms and impact concepts important to people living with CLL/SLL or MCL. Participants were encouraged to describe the experience, severity, and variation of the symptoms and impacts of disease. Cognitive debriefing was conducted to determine whether participants understood the items in the way developers intended [8, 16, 17]. All participants were debriefed on the EORTC QLQ-C30 content; specific additional items from the Item Library were then debriefed by patients in disease-specific subgroups for CLL/SLL and MCL. A combination of concurrent and retrospective verbal probing methods [16] was used for the cognitive interviewing.

#### Compliance with ethics guidelines

This mixed methods study of clinicians and patients was reviewed by WCG IRB institutional review board (IRB) and approved (Study 1,298,078). Informed consent was obtained before proceeding with clinician or patient interviews. All participants consented to have their responses included in the research and any publications reporting on the study. The study was performed in accordance with the Helsinki Declaration of 1964 and its later amendments.

#### Qualitative analysis

Verbatim de-identified patient transcripts were analyzed thematically [18] using ATLAS.ti software. A detailed line-by-line open and inductive coding approach was used [19, 20]. To ensure consistency in coding, the first two transcripts were coded independently by two researchers (JM, TL) who then met to compare their results and align on methods going forward. The codebook was revised as needed to reach coder agreement, and further developed as new concepts were identified in the remaining transcripts.

Concept data from interviews informed revision and refinement of the conceptual model. As with literature review data, Wave 1 interview codes were inductively grouped to create concepts, sub-domains, and domains to develop a visual representation of how the concepts relate to each other [18, 20, 21]. Saturation analysis was conducted sequentially per condition to assess data adequacy [22, 23], “*the point in the data collection process when no new concept-relevant information is being elicited from individual interviews or focus groups, or no new information is deemed missing during cognitive interviewing*” [24]. Saturation analysis was conducted sequentially in 5 waves of 4 transcripts for each indication; considering the granularity and conceptual overlap at the code level, saturation analysis was conducted at the sub-domain level. Cognitive debriefing analysis tabulated feedback on item appropriateness, relevance, and clarity, as well as the instructions and response scale. Hypothesized item sets from EORTC QLQ-C30 and the disease-specific library items were created, based upon the domains of the patient-informed conceptual model.

### Mixed methods: supportive quantitative strand

The newly identified item sets were then subjected to a series of quantitative analyses. These included descriptive exploratory analyses on item responses collected in Wave 1 and Wave 2, separate analyses in CLL/SLL participants and in MCL participants, and additional analyses using the full analysis set (FAS) of data including both CLL/SLL and MCL participants. These analyses were conducted on hypothesized item sets identified based upon the conceptual model generated from the qualitative research using macro-level Rasch Measurement Theory (RMT) analysis (see supplemental materials S-0^3^).

RMT analysis defines how a set of items should perform to generate reliable and valid measurements [25–27]. Exploratory RMT analysis was conducted to provide a preliminary understanding of how the hypothesized item sets performed together. The following criteria were investigated: the extent to which the items matched the sample of participants (i.e., targeting of the items to the sample), how the items covered the continuum of the measured concept, and the extent to which the item response scale worked as intended. SAS software version 9.4 was used for data preparation and description, and RUMM 2030 software was used for RMT analysis (RUMM Laboratory; Perth, Australia).

Four participants with CLL/SLL and three with MCL were excluded from the quantitative analyses because of mismatches of user IDs with the IDs used by participants in the data collected through the survey.

### Mixed methods: meta-inference

For this research, a structured approach was used to integrate the qualitative and quantitative findings, a process known as meta-inference [28]. It addressed the following criteria:

1. *Comprehensiveness of item set and targeting of instrument*.
2. *Conceptual uniqueness*.
3. *Item clarity/quality*.
4. *Appropriateness of response scale*.

## Results

### Qualitative research results

#### Literature review

After an initial search identified only two qualitative articles focused on the patient experience in MCL and CLL/SLL, we expanded the search to include NHL since qualitative research done in patients with NHL could include patients with MCL and CLL/SLL. We identified an additional 254 articles, out of which six articles were ultimately included in the analysis. Articles were excluded for the following reasons: no abstract available; not qualitative research; not patient-reported; not focused on the target conditions (MCL, CLL/SLL, or NHL); and/or no PROs or concepts provided. Key symptom concepts in both conditions identified from the literature review included fatigue, dyspnea, pain, swelling, and B symptoms such as fevers and night sweats. Key impact concepts in both conditions included functional impacts such as walking and household chores, as well as social and emotional impacts such as depression, worry, and social life limitations. Preliminary symptom and impact conceptual models for CLL/SLL and MCL were created based on the data and shared with expert clinicians for feedback (see supplemental materials S-0^5^ and S-0^6^.)

#### Clinician interviews

The clinicians participating in this study confirmed the symptoms and impacts in each disease area identified from the literature review. In CLL/SLL, clinicians highlighted that symptoms such as lymphadenopathy; symptoms associated with anemia (e.g., fatigue, shortness of breath); and systemic or B symptoms including fever, night sweats, low grade fever, and weight loss were the most noticeable at time of diagnosis (if patients were symptomatic at diagnosis) as well as at disease relapse. Clinicians described MCL as more aggressive, presenting more rapidly with more noticeable systemic symptoms (fever, night sweats, noticeable weight loss). Clinicians reported that patients usually identify fatigue as their most worrisome/problematic symptom, and that fatigue and weakness experienced lead to impacts on work and ability and desire to perform daily activities. Less clear consensus was seen in terms of the pain experience. Clinician feedback informed revision of the preliminary models of disease experience.

#### Patient interviews

Forty-one participants were enrolled and interviewed in Wave 1 (20 participants with CLL/SLL, and 21 with MCL). Participants with CLL/SLL had a mean age of 60.5 (standard deviation [SD] = 6.8) years; participants with MCL had a mean age of 62.0 (SD = 8.8) years. 70% of CLL/SLL participants and 62% of MCL participants were female. 95% of CLL/SLL participants and 90% of the MCL participants identified as White. Most participants had received prior systematic therapy (CLL/SLL 55%; MCL 90%). Table 1 summarizes participant demographic and clinical characteristics.

**Table 1:** CLL / SLL and MCL Wave 1 qualitative study demographic and clinical characteristics

Characteristic	CLL/SLL (*n* = 20)	MCL (*n* = 21)
Age		
Mean (SD), years	60.5 (6.78)	62.4 (8.8)
Sex, *n (%)*		
Female	14 (70)	13 (62)
Male	6 (30)	7 (33)
Prefer not to answer	0 (0)	1 (5)
Race/Ethnicity, *n (%)*		
White	19 (95)	19 (90)
Hawaiian Native/Pacific Islander	1 (5)	0 (0)
Black/African American	0 (0)	1 (5)
Prefer not to answer	0 (0)	1 (5)
Ethnicity, *n (%)*		
Not Hispanic or Latino	19 (95)	17 (81)
Hispanic or Latino	1 (5)	4 (19)
Education level, *n (%)*		
High school/GED	3 (15)	3 (14)
Some college	2 (10)	6 (29)
Associate degree	2 (10)	1 (5)
Bachelor’s degree	5 (25)	7 (33)
Post-graduate degree	8 (40)	3 (14)
Prefer not to answer	0 (0)	1 (5)
Comorbidities, *n (%)*^*a*^		
Arthritis	7 (35)	4 (19)
Diabetes	3 (15)	1 (5)
Heart disease	0 (0)	4 (19)
Hypertension	6 (30)	10 (48)
Obesity	2 (10)	5 (24)
Osteoporosis	3 (15)	2 (10)
Psychological stress	3 (15)	3 (14)
Treatment(s) received^b^		
Any systematic therapy	11 (55)	19 (90)
Immunotherapy	7 (35)	7 (33)
Chemoimmunotherapy	2 (10)	4 (19)
Proteosome inhibitors	1 (5)	0 (0)
Bone marrow transplant	0 (0)	1 (5)
Other	4 (20)	4 (19)
No treatment	4 (20)	0 (0)

^a^Patients could report more than one comorbidity; ^b^Patients could report more than one treatment received

**Fig. 2:**
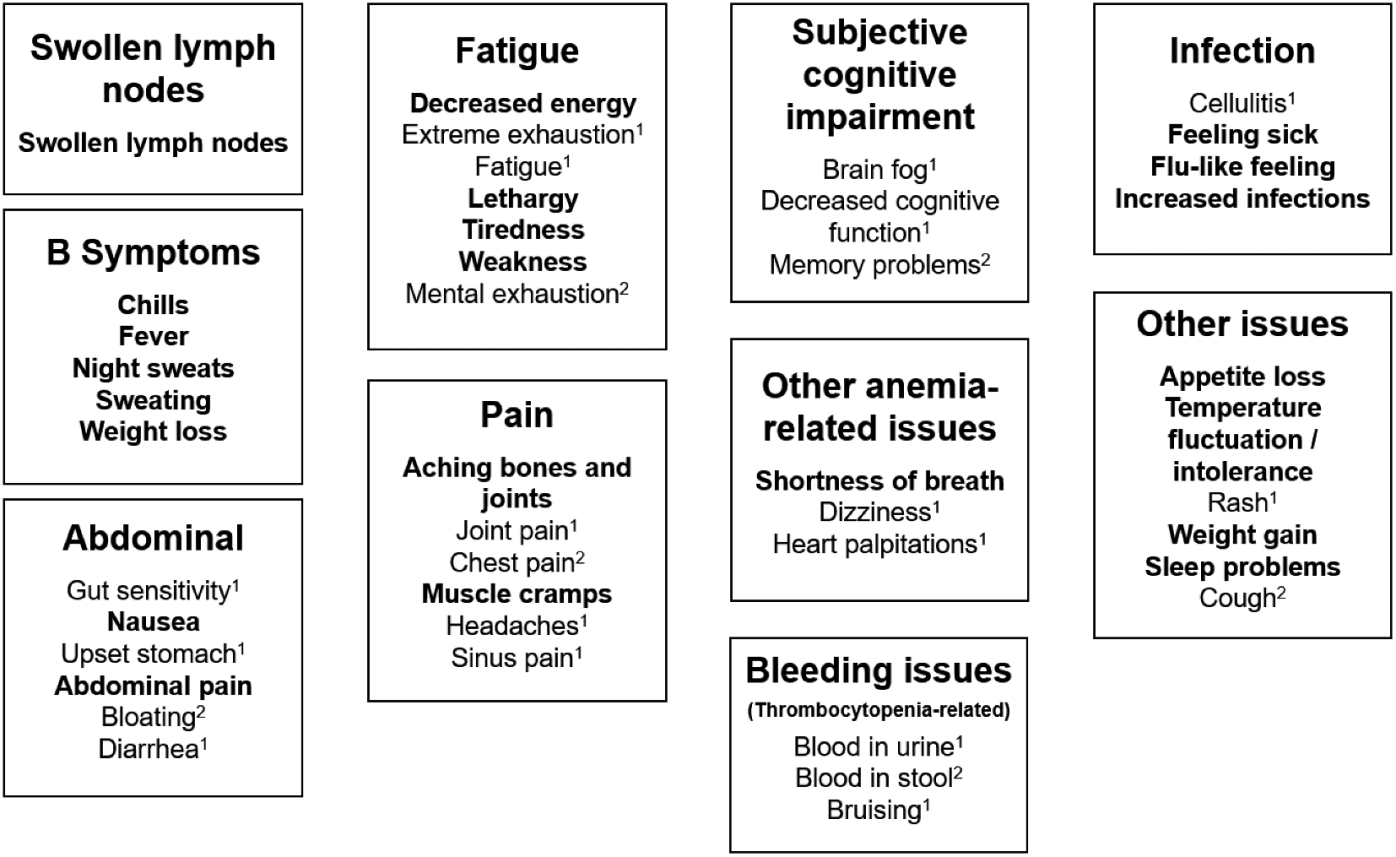
Conceptual model of CLL/SLL and MCL disease symptom experience. Bold symptoms reported in both CLL/SLL and MCL, ^1^ = CLL/SLL only, ^2^ = MCL only

**Fig. 3:**
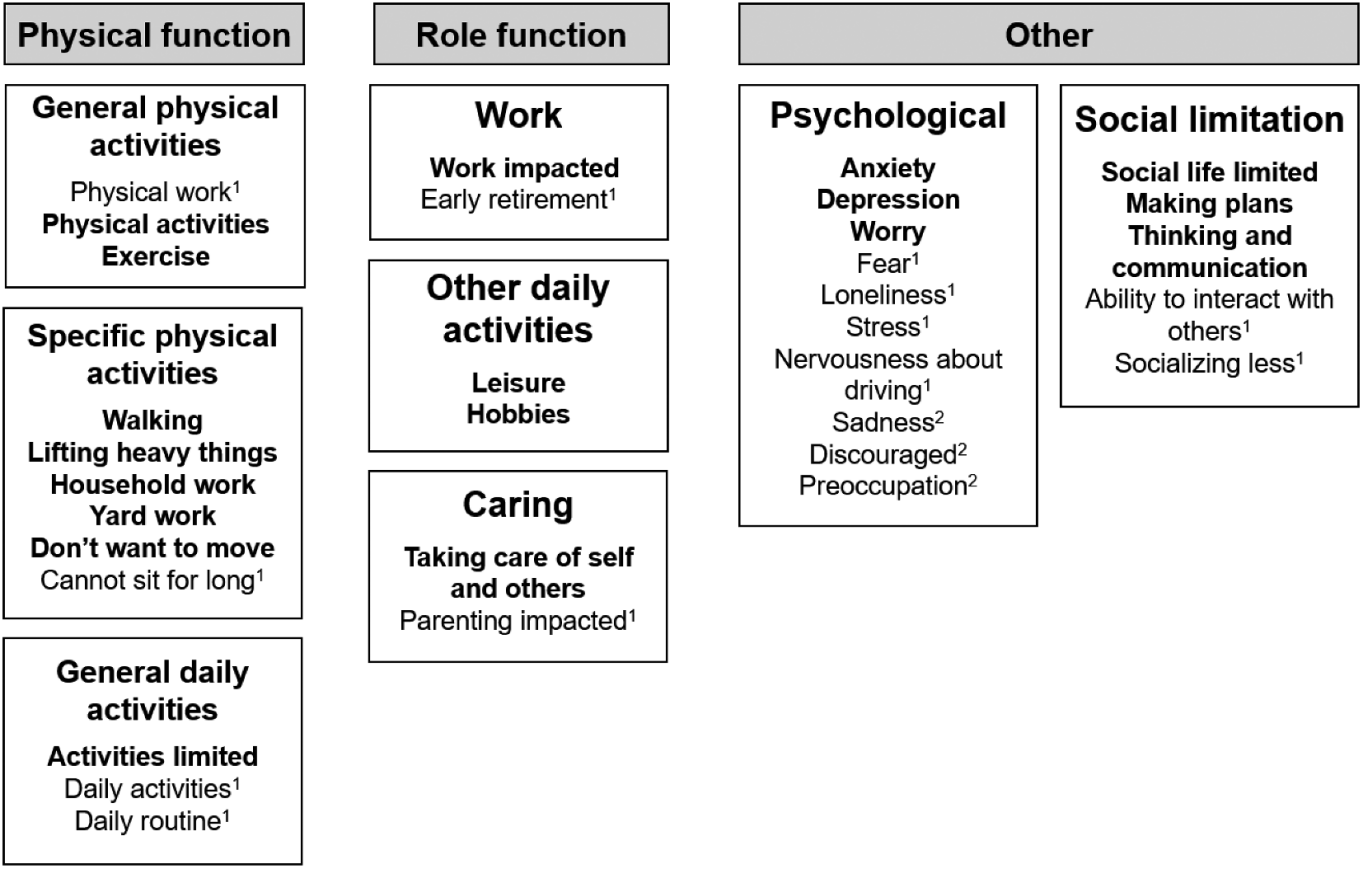
Conceptual model of CLL/SLL and MCL disease impact experience. Bold impacts reported in both CLL/SLL and MCL, ^1^ = CLL/SLL only, ^2^ = MCL only

Participants with CLL/SLL or MCL reported a variety of symptoms and disease-related impacts with a high degree of overlap among the conditions. Reported symptoms were grouped under 10 domains: swollen lymph nodes, B symptoms, abdominal issues, pain, fatigue, subjective cognitive impairment, other anemia related issues, bleeding issues related to thrombocytopenia, infection, and other issues. Impact concepts were grouped into three domains: physical function, role function, and other impacts such as psychological impact and social limitations issues. (Tables summarizing the symptom and impact domains, sub-domains, and concepts with exemplary quotes derived from CLL/SLL and MCL interviews provided in supplemental materials S-0^6^). Conceptual saturation analysis at the symptom and impact subdomain level indicated that conceptual saturation was achieved after interviewing 12 participants with CLL/SLL (i.e., the third of five groups of transcripts analyzed) and 16 participants with MCL (i.e., the fourth of five groups of transcripts analyzed). (See supplemental material S-0^7^). Figures 2 and 3 illustrate the final conceptual models for symptoms and impacts, based on the findings from literature review data, clinician insights, and qualitative data from patient interviews.

Participant interviews highlighted the importance of fatigue in people with CLL/SLL or MCL, both at diagnosis and over the course of disease. Participants described fatigue in terms of general tiredness, decreased energy, lethargy, and extreme exhaustion. The interviews with patients emphasized the persistence and pervasiveness of fatigue and its impact on everyday life. As one participant with CLL described their experience:

I had the fatigue just slowly get worse and worse. Even to this day, I have the bad fatigue, to the point that I do not want to get out of bed. I do not want to do anything, but I do. I force myself to get up and get dressed and try and do stuff, even though I don’t feel like it. I have absolutely no energy at all. And it’s been that way for about 11, 12 years. – US104.

Participants also described their experiences with B symptoms, all of which were endorsed across both CLL/SLL and MCL. Patients described experiencing these symptoms separately, successively, or in combination. As one participant with MCL explained,

You get cold, and then my low fever would start. I’d chill. I’d feel like I’m very cold. And then after that, I get my low-grade fever, and I’m warm. So that happened off and on – again, to the level that I knew I had to see what’s going on. - US140.

Concepts elicited from patient interviews in both CLL/SLL and MCL were compared with the EORTC QLQ-C30 items and the selected library items for each condition to determine whether there were gaps in conceptual coverage. Concepts addressed by the selected library items also arose from patient interviews and were included in the conceptual model. Most of the symptom and impact domains in the model were addressed by items in EORTC-QLQ-C30 and the selected library items for each condition. In both CLL/SLL and MCL, the symptom domains that were not addressed by EORTC Item Library items were swollen lymph nodes, bleeding issues, subjective cognitive impairment, and infections; the impact domain not covered was caring for self and others.

Cognitive debriefing of the EORTC QLQ-C30 and selected item sets indicated that items aligned with participants’ experience with symptoms, were well-understood, and were interpreted as intended.

Based upon the conceptual model generated from the qualitative research, four sets of items from the EORTC QLQ-C30 and the Item Library sets were identified: a fatigue set (6 items) and a B symptom set (3 items) common to both CLL/SLL and MCL, and separate disease-related symptoms sets in CLL/SLL (13 items) and MCL (13 items). (These item sets are provided in supplemental materials S-0^3^.) These sets were then used to conduct exploratory psychometric analysis.

### Quantitative research results

Since no discrepancies were discovered during the qualitative component of the Wave 1 research, the same item sets were completed in Wave 1 and Wave 2, leading to a larger sample size for the descriptive and exploratory psychometric analyses. Sixty-four participants were recruited in Wave 1 and 2, and four participants with CLL/SLL and three with MCL were excluded from the quantitative strand analysis due to data collection issues. The full analysis set (FAS) included data from 57 participants (27 with CLL/SLL and 30 participants with MCL) (Description of the demographic and health characteristics of the 27 CLL/SLL participants and the 30 MCL participants included in the FAS are available in supplemental materials S-0^8^).

The distribution of the item responses to the full EORTC QLQ-C30 questionnaire in the FAS was adequate across the four response options (i.e., all response options, from “Not at all” to “Very much”, were reported by the participants), indicating that most of the items may be relevant to the CLL/SLL and MCL participants [see supplemental materials S-0^9^, Fig. 1: Adequate distribution of the item responses to the EORTC QLQ-C30 items across the response options despite some items showing a floor effect (*FAS, n = 57)*]), especially items related to fatigue (“Need to rest”, “Felt weak”, “Were you tired”). Thirteen items showed floor or ceiling effects (i.e., when a considerable proportion of participants endorse the lowest or highest response option), indicating that these items were not able to discriminate between participants of this study at either extreme of the response scale.

The overall pattern of item responses to the CLL/SLL-related and MCL-related symptoms item sets showed an adequate distribution of the participants’ responses across the four response options, indicating that the items are able to capture the range of symptoms severity experienced by study participants, despite a possible floor effect for three items. For both CLL/SLL-related and MCL-related symptoms item sets, items measuring B symptoms, shortness of breath, and nausea suggested a possible floor effect (most CLL/SLL and MCL participants were rated in the lowest response option (i.e., “Not at all” response option), indicating that very few participants experienced these symptoms. In contrast, the responses to the items measuring fatigue included in the CLL/SLL-related symptoms and MCL-related symptoms item sets were more frequently “A little” to “Very much” (at least 65% of CLL/SLL and 53% of MCL participants), suggesting that fatigue was more frequently experienced by the participants included in this study than the other symptoms measured by the item sets (see supplemental materials S-0^9^, Fig. 2: Adequate distribution of the item responses across the response options of a) the CLL/SLL item set in the CLL/SLL participants (*n* = 27), and b) MCL item set in the MCL participants [*n* = 30]). The CLL/SLL-related symptoms and MCL-related symptoms item sets showed an adequate coverage of the severity continuum under the RMT framework (see supplemental material S-0^9^, Fig. 3: Scale to sample targeting of a) the CLL/SLL item set to the CLL/SLL participant sample (*n* = 27), and b) MCL item set to the MCL participant sample (*n* = 30)), without any major concerns in terms of fit or response scale.

The pattern of item responses to the fatigue item set in the FAS showed adequate distribution across all four response options, indicating that these items are sufficiently targeted to the study participants. The description of item responses suggested that most participants reported experiencing at least “A little” degree of symptoms as measured by four out of the six items, and fewer symptoms of “Sudden tiredness” and “Felt drowsy” (see supplemental materials S-0^9^, Fig. 4: Early outline of fatigue symptoms hierarchy in participants with CLL/SLL and MCL [*Fatigue item set; FAS, n = 57*]). The fatigue item set also showed good coverage of the severity continuum under the RMT framework, without any major concerns in terms of fit or response scale. The exploration of this item set also provided an early outline of the fatigue symptom hierarchy, from the less severe (“Were you tired”) to the most severe (“Felt drowsy”) (see supplemental material S-0^9^, Fig. 5: Most CLL/SLL and MCL participants did not experience B symptoms [*FAS, n = 57*]).

Finally, the B symptoms item set showed a major floor effect: more than half of the participants from the FAS reported “Not at all” to the three B symptoms (see supplemental materials S-0^9^, Fig. 5: Most CLL/SLL and MCL participants did not experience B symptoms [*FAS, n = 57*]), indicating that most participants reported not experiencing any of these three symptoms in the week preceding the survey completion. Additionally, the RMT analysis indicated that the response scale of B symptoms items may be problematic.

### Meta-inference

Qualitative findings indicated the importance of fatigue to participants in both CLL/SLL and MCL. Fatigue manifestations were the most frequently reported by qualitative study participants and appeared first in the early outline of the CLL/SLL-related and MCL-related symptom hierarchy. The fatigue item set showed promising results for the measurement of fatigue manifestations in CLL/SLL and MCL using the selected items, supporting the qualitative findings.

Questionnaire responses indicated that B symptoms were experienced by only a minority of the participants at the time of the study and may not form a consistent scale. These items covered a small range of the participants, with a major floor effect. However, qualitative findings in both CLL/SLL and MCL indicated that these symptoms are clinically relevant and meaningful to participants and thus important to retain. It may be better to consider the three B symptoms independently as single items, rather than calculating a B symptoms score. Alternately, including the three B symptoms in the overall CLL/SLL-related and MCL-related symptoms item sets and symptom scores may be acceptable. Analysis in a larger sample size is needed to better understand this finding.

Finally, qualitative findings indicated that the CLL/SLL- and MCL-specific items were well understood, relevant, and accepted by participants, though one or two participants per sample flagged not having experienced specific symptoms such as sudden tiredness, appetite loss, fever/chills, night sweats, nausea, and bloating in abdomen. Both clinician and patient interviews indicated that shortness of breath (particularly at exertion) was a relevant aspect of the patient experience, but rarely progressed from shortness of breath to a feeling of being breathless. Preliminary supportive quantitative analysis of the CLL/SLL- and MCL-related symptom item sets confirmed that the items were well targeted to the symptoms experienced by the participants and fit the Rasch model, suggesting that creating scores combining these items is reasonable. Finally, the quantitative analysis also provided early insight into the severity continuum of disease. In CLL/SLL, this continuum ranged from fatigue symptoms, then B symptoms, then dyspnea; in MCL, the continuum ranged from fatigue symptoms to generic pain, then B symptoms, dyspnea, nausea, and bloated feeling in abdomen). In sum, findings supported the importance of symptoms related to fatigue and anemia.

## Discussion

This mixed methods study provides preliminary evidence to support the selection of items from the EORTC Item Library to evaluate fatigue, B symptoms, and CLL/SLL- and MCL-specific symptoms. The targeted literature search, clinician interviews, and patient interviews were used to explore disease symptoms and symptom-related impacts from the patient perspective. The qualitative work resulted in the identification of conceptual models of disease symptoms and symptom-related impacts that provide preliminary evidence supporting the content validity of the items compiled from the EORTC Item Library. Exploratory RMT analysis provides initial evidence that supports the use of these item sets to evaluate patient outcomes.

The literature review conducted to support this study highlighted a dearth of patient-focused, qualitative data in CLL, SLL, and MCL. The qualitative research conducted with patient participants contributes to the literature on the patient experience of these conditions.

The three items from the EORTC Item Library pre-selected to capture B symptoms for both CLL/SLL and MCL (fever/chills, night sweat, loss of appetite) were all endorsed as relevant by the participants in the patient interviews in all three conditions, though quantitative assessment indicated these symptoms were experienced by a minority of participants. Of note, B symptoms are more likely to be present in CLL/SLL and MCL patients when the disease is progressing or when symptoms are not well-controlled [29]. Since there is no clear evidence supporting the creation of a continuum of overall severity of B symptoms, the question of whether or not to group these symptoms together remains. It may be preferable to consider the three B symptoms independently using single items rather than calculating a composite B symptoms score. However, given their relevance to CLL/SLL and MCL participants, including them in overall CLL/SLL-related and MCL-related symptom scores may be an acceptable alternative. This question is particularly challenging given that only a minority of patients endorsed these three items related to B symptoms in the quantitative part of this study. Finally, it should be noted that for one of the B symptoms, weight loss (typically defined as “weight loss of > 10% of normal body weight over a period of 6 months or less” [30]), our research tentatively used an imperfect proxy for the corresponding PRO item, “lack of appetite.” The reason for this was that weight loss is an objective sign (not a symptom) that does not require a patient report to be measured. For this reason, the only PRO item that could be related to it would have been “loss of appetite.” The issue is that this item captured a different concept. However, as it was endorsed by the participants in the qualitative component of the research, it was kept in the final set of items.

Of note, though swollen lymph nodes were reported by both participants with CLL/SLL and with MCL, no item specific to swollen lymph nodes was available in the EORTC Item Library; therefore, this domain was not included in the PRO items. This may be a gap that the EORTC would like to address in their future development to add an item to assess swollen lymph nodes in their Item Library.

This research contributes to the growing body of literature supporting the pragmatic use of targeted item sets from EORTC QLQ-C30 and the EORTC Item Library to cover condition-specific concepts in hematologic malignancies [12–14, 31]. This approach is especially important, as the QLQ-C30 plus a static, disease-specific questionnaire module alone may not always be adequate or fit-for-purpose as treatment modalities evolve [32]. The flexibility of adding additional targeted items to capture aspects of disease and treatment not previously considered allows researchers to collect a more nuanced assessment of patient experience.

While this research generated preliminary data for consideration in the identification of measures to assess PROs among patients with CLL/SLL or MCL, some limitations must be acknowledged. Firstly, the study relied on patient-reported diagnoses, and patients did not report on whether they presented with classic MCL (cMCL) or the leukemic non-nodal/indolent variant of MCL (L-NN MCL). Secondly, while the participants represented an adequate range in terms of age, and a good range of education levels, the study population was mostly White and non-Hispanic, which limits the generalizability of findings to patients of other races and ethnicities. It is also notable that 62% of the participants with MCL were women. Given that MCL predominantly impacts men, this facet of our sample may constitute a limitation on external validity, and future research is warranted. Thirdly, the expansion of recruitment criteria to include participants who were on a longer remission may have impacted the accuracy of participants’ recall memory and introduced more variability in their experiences of these symptoms. Participants who had been in remission for a longer duration may have difficulty recalling specific details about their symptoms during the more active phase of their cancer. The qualitative results reflect the fact that participants were thinking about the entire cancer experience, some significant symptoms during active disease such as fevers/chills or night sweats (questions in B symptom item set) may be still in their recall memory. However, when participants in remission responded to the EORTC item sets, which was limited to 7-day recall period, in the quantitative portion of this study, they would be less likely to report B symptoms. Nevertheless, the study collected rich qualitative data reflecting a wide range of symptoms and impacts. The last limitation pertains to the sample size with only data from 57 participants (27 with CLL/SLL and 30 with MCL) were available for the quantitative analyses. Thus, it must be emphasized that the quantitative analysis was only intended to provide early insights on the item sets. Given the small sample size, there is uncertainty on the parameter estimates from the Rasch model, which limits the interpretation of these quantitative results. While RMT has been shown to provide robust results in small samples [33, 34], the results from this study should be interpreted cautiously; further research is needed to better understand the psychometric properties of the proposed item sets and generate the necessary evidence to support their use in these patient populations.

## Conclusions

This mixed-methods study combining qualitative research with quantitative psychometric analyses found that the sets of selected items from the EORTC Item Library may be useful measures to evaluate endpoints reflecting disease-related symptoms in clinical trials in CLL/SLL or MCL. The psychometric findings from this research are preliminary, and the PRO measures resulting from this mixed methods research should undergo additional psychometric evaluation in larger patient cohorts to be considered fully fit-for-purpose for the demonstration of treatment benefits in these populations.

### Electronic supplementary material

Below is the link to the electronic supplementary material.

Supplementary Material 1: Search strategy

Supplementary Material 2: Inclusion criteria

Supplementary Material 3: CLL/SLL - MCL item sets

Supplementary Material 4: Literature search results

Supplementary Material 5: Conceptual model evolution

Supplementary Material 6: Concept summary tables

Supplementary Material 7: Saturation analysis tables

Supplementary Material 8: Full analysis set description

Supplementary Material 9: Selected quantitative outputs

## Data Availability

The datasets generated during and/or analyzed during the current study are available from the corresponding author on reasonable request.

## Declarations

### Editorial assistance

Editorial assistance was provided by Sara Strzok and Trais Pearson, PhD of Modus Outcomes.

## References

[1] Burger JA, Ghia P, Rosenwald A, Caligaris-Cappio F (2009). The microenvironment in mature B-cell malignancies: a target for new treatment strategies. Blood the Journal of the American Society of Hematology.

[2] Burger JA, Tedeschi A, Barr PM, Robak T, Owen C, Ghia P (2015). Ibrutinib as initial therapy for patients with chronic lymphocytic leukemia. N Engl J Med.

[3] Isaac K, Mato AR (2020). Acalabrutinib and its therapeutic potential in the treatment of chronic lymphocytic leukemia: a short review on Emerging Data. Cancer Manag Res.

[4] Syed YY, Zanubrutinib (2020). First Approval Drugs.

[5] Wierda WG, Lewis DJ, Ghia P, Shah NN, Coombs CC, Cheah CY (2022). Efficacy of Pirtobrutinib, a highly Selective, non-covalent (Reversible) BTK inhibitor in Richter Transformation: results from the phase 1/2 BRUIN study. Blood.

[6] United States Food and Drug Administration (2021) Core patient-reported outcomes in cancer clinical trials: guidance for industry [draft guidance].

[7] United States Food and Drug Administration (2018) Patient-focused drug development: collecting comprehensive and representative input. Guidance for industry.

[8] U.S. Food and Drug Administration. Patient-focused drug development: methods to identify what is important to patients 2019 Available from: https://www.fda.gov/media/131230/download

[9] Oerlemans S, Efficace F, Kieffer JM, Kyriakou C, Xochelli A, Levedahl K (2022). International validation of the EORTC QLQ-CLL17 questionnaire for assessment of health‐related quality of life for patients with chronic lymphocytic leukaemia. Br J Haematol.

[10] van de Poll-Franse L, Oerlemans S, Bredart A, Kyriakou C, Sztankay M, Pallua S (2018). International development of four EORTC disease-specific quality of life questionnaires for patients with Hodgkin lymphoma, high-and low-grade non-hodgkin lymphoma and chronic lymphocytic leukaemia. Qual Life Res.

[11] Piccinin C, Basch E, Bhatnagar V, Calvert M, Campbell A, Cella D (2023). Recommendations on the use of item libraries for patient-reported outcome measurement in oncology trials: findings from an international, multidisciplinary working group. Lancet Oncol.

[12] Bell JA, Galaznik A, Pompilus F, Strzok S, Bejar R, Scipione F (2019). A pragmatic patient-reported outcome strategy for rare disease clinical trials: application of the EORTC item library to myelodysplastic syndromes, chronic myelomonocytic leukemia, and acute myeloid leukemia. J patient-reported Outcomes.

[13] Regnault A, Pompilus F, Ciesluk A, Mazerolle F, Bejar R, Fram RJ (2021). Measuring patient-reported physical functioning and fatigue in myelodysplastic syndromes using a modular approach based on EORTC QLQ-C30. J patient-reported Outcomes.

[14] Barrett L, Elliott E, Voorhaar M, Ingelgård A, Griebsch I, Wong B (2023). A mixed-methods study to better measure patient-reported Pain and fatigue in soft tissue sarcoma. Oncol Ther.

[15] Kulis D, Bottomley A, Whittaker C, van de Poll-Franse L, Darlington A, Holzner B (2017). The use of the EORTC item library to supplement EORTC quality of life instruments. Value in Health.

[16] Willis GB, Artino AR (2013). What do our respondents think we’re asking? Using cognitive interviewing to improve medical education surveys. J Graduate Med Educ.

[17] Brod M, Tesler LE, Christensen TL (2009). Qualitative research and content validity: developing best practices based on science and experience. Qual Life Res.

[18] Bryman A, Burgess (2002). B. analyzing qualitative data.

[19] Thomas DR (2006). A General Inductive Approach for analyzing qualitative evaluation data. Am J Evaluation.

[20] Bowling A (2009). Research methods in Health: Investigating Health and Health Services.

[21] Klassen A, Pusic A, Scott A, Klok J, Cano S (2009). Satisfaction and quality of life in women who undergo breast surgery: a qualitative study. BMC Womens Health.

[22] Morse JM (1995) The significance of saturation. Sage Publications Sage CA: Thousand Oaks, CA; p. 147-9

[23] Hennink M, Kaiser BN (2022). Sample sizes for saturation in qualitative research: a systematic review of empirical tests. Soc Sci Med.

[24] Rothman M, Burke L, Erickson P, Leidy NK, Patrick DL, Petrie CD (2009). Use of existing patient-reported outcome (PRO) instruments and their modification: the ISPOR Good Research practices for evaluating and documenting content validity for the use of existing instruments and their modification PRO Task Force Report. Value in Health.

[25] Andrich D, de Jong JHAL, Sheridan BE, Rost J, Langeheine R (1997). Diagnostic opportunities with the Rasch model for ordered response categories. Applications of latent trait and latent class models in the saocial sciences.

[26] Andrich D, Luo G, Sheridan BE, Interpreting (2012). RUMM2030 part IV: multidimensionality and subtests in RUMM.

[27] Andrich D, Sheridan BRUMM (2030) Perth, WA: RUMM Laboratory Pty Ltd; 1997–2014

[28] Regnault A, Willgoss T, Barbic S (2017). Towards the use of mixed methods inquiry as best practice in health outcomes research. J Patient Rep Outcomes.

[29] Rozovski U, Keating MJ, Estrov Z (2013). Targeting inflammatory pathways in chronic lymphocytic leukemia. Crit Rev Oncol Hematol.

[30] Rosenberg SA, Boiron M, DeVita VT, Johnson RE, Lee BJ, Ultmann JE (1971). Report of the committee on Hodgkin’s disease staging procedures. Cancer Res.

[31] Ciesluk A, Voorhaar M, Barrett L, Baldasaro J, Griebsch I, Marquis P (2022). Measuring the patient experience in Rare disorders: benefit of pragmatic mixed-methods research in NUT carcinoma. Oncol Ther.

[32] EORTC Quality of Life. Item library — The new QLG strategy: core + module + Item Library Available from: https://qol.eortc.org/item-library/

[33] Azizan NH, Mahmud Z, Rambli A (2020). Rasch rating scale item estimates using maximum likelihood approach: effects of sample size on the accuracy and bias of the estimates. Int J Adv Sci Technol.

[34] Linacre J (1994) Sample size and item calibration stability. Rasch Meas Trans.; 7: 328. 2016

