# Supplementary material for: Mixed-methods research to support the use of new lymphoma-specific patient-reported symptom measures derived from the EORTC item library
Source: J Patient Rep Outcomes. 2024 Jan 22;8:8. doi: 10.1186/s41687-024-00683-2 (PMC10803695; doi:10.1186/s41687-024-00683-2)
Supplement: Supplementary file 1 — Supplementary Material 1: Search strategy [file 41687_2024_683_MOESM1_ESM.docx]

#### S-01 Search strategy for CLL/SLL

Search terms were developed for PubMed (Table 1) to determine an adequate and comprehensive strategy. Articles describing patient-reported, qualitative data regarding the experience of CLL/SLL symptoms and impacts from the perspective of patients were targeted.

Articles were screened using a two-stage process, first by title/abstract, and then by reviewing the full text. Articles were included for full-text review and data extraction if they discussed patient-reported concepts related to the patient experience of CLL or SLL.

Table 1. CLL/SLL concept literature search strategy; dated May 13, 2020

| Search | Term(s) | Results |
| --- | --- | --- |
| 1 | Qualitative OR interview OR focus group OR survey OR framework | 1,958,890 |
| 2 | Chronic lymphocytic leukemia OR small lymphocytic lymphoma | 26,138 |
| 3 | 1 AND 2 | 561 |
| 4 | Filters: Humans, English | 498 |

Search strategy for MCL

Search terms were developed for PubMed (Table 1) to determine an adequate and comprehensive strategy. Articles describing patient-reported, qualitative data regarding the experience of MCL symptoms and impacts from the perspective of patients were targeted. In the event that no data was found for MCL, we planned to conduct a secondary search for concepts that describe the patient experience of non-Hodgkin’s lymphoma (NHL) (Table 2).

Articles were screened using a two-stage process, first by title/abstract, and then by reviewing the full text. Articles were included for full-text review and data extraction if they discussed patient-reported concepts related to the patient experience of MCL or NHL.

*Table 1. MCL concept literature search strategy; dated May 13, 2020.*

| Search | Term(s) | Results |
| --- | --- | --- |
| 1 | Qualitative OR interview OR focus group OR survey OR framework | 1,958,890 |
| 2 | mantle cell lymphoma | 5,727 |
| 3 | 1 AND 2 | 116 |
| 4 | Filters: Humans, English | 103 |

*Table 2. NHL concept literature search strategy; dated August 24, 2020*

| Search | Term(s) | Results |
| --- | --- | --- |
| 1 | Qualitative OR interview OR focus group OR survey OR framework [Field: Title/Abstract] | 1,094,295 |
| 2 | Non-Hodgkin's lymphoma [Field: Title/Abstract] | 19,639 |
| 3 | 1 AND 2 | 293 |
| 4 | Filters: Humans, English | 254 |
