# Supplementary material for: Mixed-methods research to support the use of new lymphoma-specific patient-reported symptom measures derived from the EORTC item library
Source: J Patient Rep Outcomes. 2024 Jan 22;8:8. doi: 10.1186/s41687-024-00683-2 (PMC10803695; doi:10.1186/s41687-024-00683-2)
Supplement: Supplementary file 2 — Supplementary Material 2: Inclusion criteria [file 41687_2024_683_MOESM2_ESM.docx]

S-02 Inclusion/Exclusion criteria for CLL/SLL

The recruitment partner screened interested participants by asking them eligibility questions. Patients were asked to provide documentation to the recruitment agency to verify eligibility, including evidence of diagnosis (confirmation of diagnosis from doctor and/or lab work). The type of evidence was documented in the screener form.

*Inclusion criteria*

The initial inclusion criteria were as follows:

1. The patient provides written consent to participate in this research
2. The patient speaks, reads, and understands English
3. The patient is at least 18 years old
4. The patient is willing to participate in one or two audio-recorded telephone interviews lasting approximately 60 to 90-minutes each
5. The patient has a clinically confirmed diagnosis of R/R or frontline CLL/SLL (as described above)

*Exclusion criteria*

The initial exclusion criteria were as follows:

1. The patient is currently in remission (i.e., absence of active CLL/SLL, MCL, or other NHL for at least one month or greater)
2. The patient has a history of cancer diagnosis or has been treated for a different form of cancer (other than CLL, SLL, MCL, or other NHL) within the last 5 years, not including diffuse large B-cell lymphoma, or non-melanoma skin cancer
3. The patient has a diagnosis of both CLL/SLL and MCL
4. The patient has any visual, auditory, cognitive, or linguistic impairment that would prevent him/her from understanding and answering the questions of the interviewer at the time of the interview

Following recruitment difficulties (10 patients recruited after approximately four months), the exclusion criteria were changed to the following:

1. The patient has been in remission (i.e., absence of active CLL, SLL, MCL, or other NHL) for more than 12 months
2. The patient has a history of cancer diagnosis or has been treated for a different form of cancer (other than CLL, SLL, MCL, or other NHL) within the last 5 years, not including diffuse large B-cell lymphoma, or non-melanoma skin cancer
3. The patient has a diagnosis of both CLL/SLL and MCL
4. The patient has any visual, auditory, cognitive, or linguistic impairment that would prevent him/her from understanding and answering the questions of the interviewer at the time of the interview

Inclusion/Exclusion criteria for MCL

The recruitment partner screened interested participants by asking them eligibility questions. Patients were asked to provide documentation to the recruitment agency to verify eligibility, including evidence of diagnosis (confirmation of diagnosis from doctor, lab work). The type of evidence was documented in the screener form.

*Inclusion criteria*

The initial inclusion criteria were as follows:

- - 1. The patient provides written consent to participate in this research
    2. The patient speaks, reads, and understands English
    3. The patient is at least 18 years old
    4. The patient is willing to participate in one or two audio-recorded telephone interviews lasting approximately 60 to 90-minutes each
    5. The patient has a clinically confirmed diagnosis of R/R MCL (as described above)

*Exclusion criteria*

The initial exclusion criteria were as follows:

1. The patient is currently in remission (i.e., absence of active CLL/SLL, MCL, or other NHL for at least one month or greater)
2. The patient has a history of cancer diagnosis or has been treated for a different form of cancer (other than CLL, SLL, MCL, or other NHL) within the last 5 years, not including diffuse large B-cell lymphoma, or non-melanoma skin cancer
3. The patient has a diagnosis of both CLL/SLL and MCL
4. The patient has any visual, auditory, cognitive, or linguistic impairment that would prevent him/her from understanding and answering the questions of the interviewer at the time of the interview

Following recruitment difficulties (2 patients recruited after approximately four months), the inclusion criteria were updated to include frontline MCL, and the exclusion criteria were changed to the following:

1. The patient has been in remission (i.e., absence of active CLL, SLL, MCL, or other NHL) for more than 12 months
2. The patient has a history of cancer diagnosis or has been treated for a different form of cancer (other than CLL, SLL, MCL, or other NHL) within the last 5 years, not including diffuse large B-cell lymphoma, or non-melanoma skin cancer
3. The patient has a diagnosis of both CLL/SLL and MCL
4. The patient has any visual, auditory, cognitive, or linguistic impairment that would prevent him/her from understanding and answering the questions of the interviewer at the time of the interview
