# Supplementary material for: Mixed-methods research to support the use of new lymphoma-specific patient-reported symptom measures derived from the EORTC item library
Source: J Patient Rep Outcomes. 2024 Jan 22;8:8. doi: 10.1186/s41687-024-00683-2 (PMC10803695; doi:10.1186/s41687-024-00683-2)
Supplement: Supplementary file 3 — Supplementary Material 3: CLL/SLL - MCL item sets [file 41687_2024_683_MOESM3_ESM.docx]

#### S-03 CLL/SLL and MCL item sets

Hypothesized item sets were identified based upon the conceptual model generated from the qualitative research strand. These included a fatigue item set, a B symptom item set, a CLL/SLL symptom item set, and an MCL symptom item set.

Table 1. Fatigue item set

| Item | Source |
| --- | --- |
| Did you need to rest? | QLQ-C30 |
| Have you felt weak? | QLQ-C30 |
| Were you tired? | QLQ-C30 |
| Have you had a lack of energy? | Item library |
| Have you felt drowsy? | Item library |
| Have you had sudden tiredness? | Item library |

Table 2. B symptom item set

| Item | Source |
| --- | --- |
| Have you lacked appetite? | QLQ-C30 |
| Have you had night sweats? | Item library |
| Have you had fevers or chills? | Item library |

Table 3. CLL/SLL item set

| Item | Source |
| --- | --- |
| Did you need to rest? | QLQ-C30 |
| Have you felt weak? | QLQ-C30 |
| Were you tired? | QLQ-C30 |
| Have you had trouble sleeping? | QLQ-C30 |
| Have you had pain? | QLQ-C30 |
| Were you short of breath? | QLQ-C30 |
| Have you felt nauseated? | QLQ-C30 |
| Have you lacked appetite? | QLQ-C30 |
| Have you had a lack of energy? | Item library |
| Have you felt drowsy? | Item library |
| Have you had sudden tiredness? | Item library |
| Have you had night sweats? | Item library |
| Have you had fevers or chills? | Item library |

Table 4. MCL item set

| Item | Source |
| --- | --- |
| Did you need to rest? | QLQ-C30 |
| Have you felt weak? | QLQ-C30 |
| Were you tired? | QLQ-C30 |
| Have you had pain? | QLQ-C30 |
| Were you short of breath? | QLQ-C30 |
| Have you felt nauseated? | QLQ-C30 |
| Have you lacked appetite? | QLQ-C30 |
| Have you had a lack of energy? | Item library |
| Have you felt drowsy? | Item library |
| Have you had sudden tiredness? | Item library |
| Have you had night sweats? | Item library |
| Have you had fevers or chills? | Item library |
| Have you had a bloated feeling in your abdomen | Item library |
