# Supplementary material for: Mixed-methods research to support the use of new lymphoma-specific patient-reported symptom measures derived from the EORTC item library
Source: J Patient Rep Outcomes. 2024 Jan 22;8:8. doi: 10.1186/s41687-024-00683-2 (PMC10803695; doi:10.1186/s41687-024-00683-2)
Supplement: Supplementary file 4 — Supplementary Material 4: Literature search results [file 41687_2024_683_MOESM4_ESM.docx]

S-04 CLL/SLL concept literature review

When the search was conducted on June 8, 2020, 540 articles were identified on PubMed; however, the majority were screened out at the abstract level – most because they were not qualitative; nine articles were reviewed at the full-text level, and two were included in the analysis. Figure 1 summarizes search results in PRISMA format. Table 1 summarizes data extracted from the relevant articles.


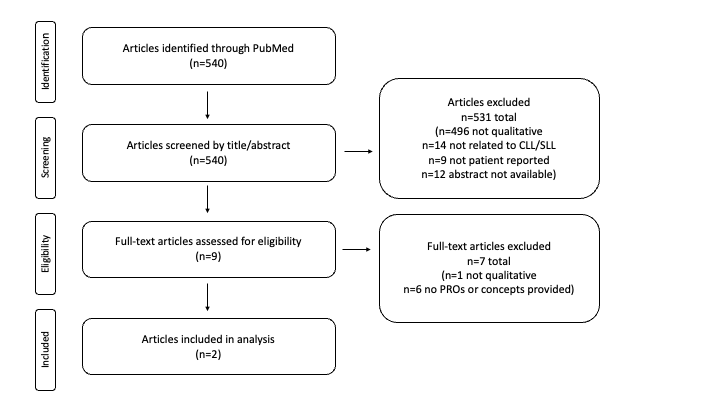


*Figure 1. CLL/SLL concept search PRISMA diagram*

*Table 1. Summary results of full-text articles for CLL/SLL*

| First author | Title | Year | Journal | Sample | Methods | Type of analysis | Concepts |
| --- | --- | --- | --- | --- | --- | --- | --- |
| McCarrier | Concept elicitation within patient-powered research networks: a feasibility study in chronic lymphocytic leukemia | 2016 | Value in Health | N=50 | Open-ended web survey and follow-up qualitative telephone interviews | Interviews were coded using Atlas.ti and SPSS was used for descriptive analyses of categorical survey response variables. | Symptoms: Allergy/rash, bruising, cognitive difficulties, swelling in feet/ankles.  Impacts: irritability, shock, difficulty staying asleep, use of medical additives, and management of priorities. |
| Evans | Incurable, invisible and inconclusive: watchful waiting for chronic lymphocytic leukemia and implications for doctor-patient communication | 2012 | European Journal of Cancer Care | N=12 | Qualitative interviews | Thematic analysis using Nvivo7, sentences and paragraphs from each interview transcript were allocated to codes that reflected anticipated and emergent themes using the qualitative method of constant comparison. | Symptoms: Swollen lymph nodes, chest infections, sensitivity to insect bites and stings, cramps, joint aches, breathlessness, fatigue, gut sensitivity, hemolysis, run down, vulnerability to infection, poor sleep, sweats, wounds, slow to heal, lack of energy, some reported being asymptomatic  Impacts: retired early causing loss of identity, difficulty coming to terms with diagnosis, traveling became tiring, avoiding contact with others to avoid infections, going out less, socializing less, feeling of isolation, having to manage symptoms, difficulty doing household chores, gardening, Invisible condition, lifestyle adaptations |

MCL concept literature review

When the MCL search was conducted on June 8, 2020, 113 articles were identified on PubMed; however, all were screened out at the abstract level – most because they were not qualitative. See Figure 2 for PRISMA diagram. Because there were no articles found, we ran the NHL search on August 24, 2020. Although 254 articles were identified, the majority (n=247) were excluded at the full-text level, because most were not qualitative papers. Seven full-text articles were assessed for eligibility and two full-text articles were also added from a previously conducted literature review (found from Google Scholar search), resulting in six articles included in the analysis. See Figure 2 for PRISMA diagram and Table 4 for a summary of the results.


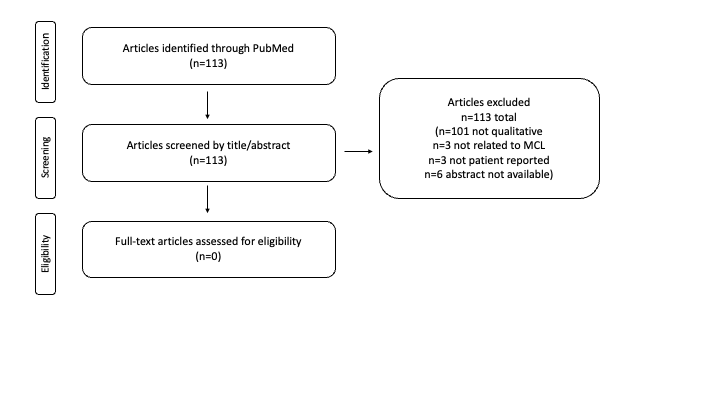


*Figure 2. MCL concept search PRISMA diagram*


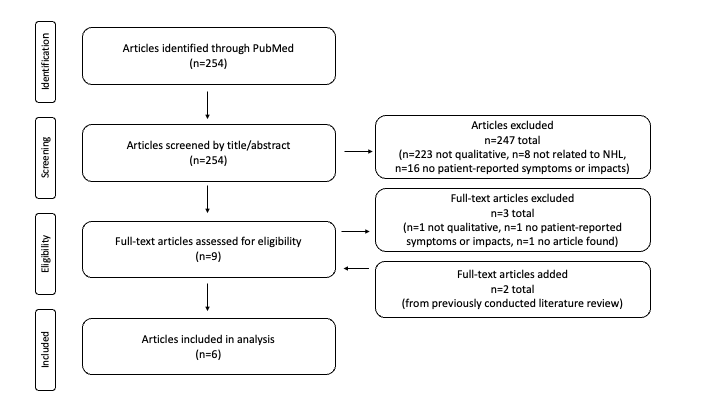


*Figure 3. NHL concept search PRISMA diagram*

*Table 4. Summary results of full-text articles for NHL*

| First author | Title | Year | Journal | Sample | Methods | Type of analysis | Concepts |
| --- | --- | --- | --- | --- | --- | --- | --- |
| Chircop | The lived experience of patients with non-Hodgkin’s lymphoma undergoing chemotherapy | 2018 | European Journal of Oncology Nursing | N=6 adult patients with NHL undergoing chemotherapy | Semi-structured interviews | Interpretative phenomenological analysis | Living an emotional rollercoaster - the emotional experience patients have including fear of the unknown, relief at commencing treatment, overwhelmed as the side effects of chemo hit Becoming dependent on others - physical impact of the treatment, resulting in becoming less active and more dependent on others for support and care, acceptance that this is their destiny, upset at not being able to maintain their daily routine, being at the mercy of your body Facing an uncertain future - how the participants viewed the future, during which they would undergo more chemotherapy, ruminating about what lies ahead, reverting back to 'normal' |
| Chircop | Being diagnosed with cancer: The experiences of patients with non-Hodgkin's lymphoma | 2017 | Journal of Clinical Nursing | Six adult patients with NHL | Two semi-structured interviews at different timepoints | Interpretative phenomenological analysis | Emotional reaction to the diagnosis  Struggling with a lack of understanding Searching through past experiences Most participants expressed shock, disbelief and fear at the news of their diagnosis |
| Chircop | Coping with non-Hodgkin's lymphoma: a qualitative study of patient perceptions and supportive care needs whilst undergoing chemotherapy | 2017 | Supportive Care in Cancer | Six adult patients with NHL receiving chemotherapy | Two semi-structured interviews | Interpretative phenomenological analysis | Interpreting life through a different lens -creating short term goals, experiencing spiritual growth Wearing a mask - keeping up an appearance, a shield from emotional distress Suppressing thoughts about chemotherapy -  thoughts about cancer trajectory and physical side effects experienced Support along the journey - support from health care providers, support from family and friends |
| Swash | 'Haematological cancers, they're a funny bunch': A qualitative study of non-Hodgkin's lymphoma patient experiences of unmet supportive care needs | 2018 | Journal of Health Psychology | n=6 NHL patients, located in the UK; at least 18 months post-diagnosis; completed treatment | Focus groups to explore patient needs during diagnosis, treatment, and survivorship | Qualitative | Physical impact, practical impact, adjusting, perception of self, psychological need, need to feel supported, social support, medical support, psychological support, psychosocial concerns, control |
| Wall | Experiences prior to diagnosis of non-Hodgkin lymphoma: a phenomenological study | 2011 | Journal of Advanced Nursing | N=31 | Qualitative interviews to identify the patients’ experience leading to diagnosis | Phenomenological approach, in-depth qualitative interviews, open-ended questions | Symptoms: swelling (neck, breast), acute or long-term illness, lethargy, urinary infection, migraine, cough, itching, breathlessness, night sweats, pain, spleen enlarged, influenza, infections  Impacts: Worries, anxiety, fear, panic |
| Howell | Help-seeking behavior in patients with lymphoma | 2008 | European Journal of Cancer Care | N=32 lymphoma patients (91% with NHL; of these, 31% had diffuse large B-cell lymphoma, and 31% had follicular lymphoma) | Semi-structured qualitative interviews to identify the beliefs and actions of lymphoma patients seeking help for symptoms | Qualitative coding | Lump, nodule, gland, swelling, tired, fatigue, lethargic, weak, no energy, couldn’t eat, unable to eat much, soon full, no appetite, indigestion, sweating, hot sweats, shivering, cold shakes, skin irritation, itching, eczema, scratching, losing weight, thin and frail, couldn’t walk as well, up steps, up slopes, legs went, swollen ankle, feet, leg (no injury), pain,  stomach-ache, stomach blown up, bloated, chest infection, flu, cold, cough, breathless, bladder frequency, kidney infections, feeling sick, nausea, swallowing difficulties, thirsty, dry mouth and lips, stiff feeling in neck, difficult to turn head, fractious, nerves, anaemia, pallor, couldn’t drink, vomiting blood, constipated, sore throat, croaking voice, couldn’t lift arms, palpitations, hair loss |
