# Supplementary material for: Mixed-methods research to support the use of new lymphoma-specific patient-reported symptom measures derived from the EORTC item library
Source: J Patient Rep Outcomes. 2024 Jan 22;8:8. doi: 10.1186/s41687-024-00683-2 (PMC10803695; doi:10.1186/s41687-024-00683-2)
Supplement: Supplementary file 5 — Supplementary Material 5: Conceptual model evolution [file 41687_2024_683_MOESM5_ESM.docx]

S-05 Preliminary conceptual models

Conceptual models in CLL/SLL and NHL developed following literature review.


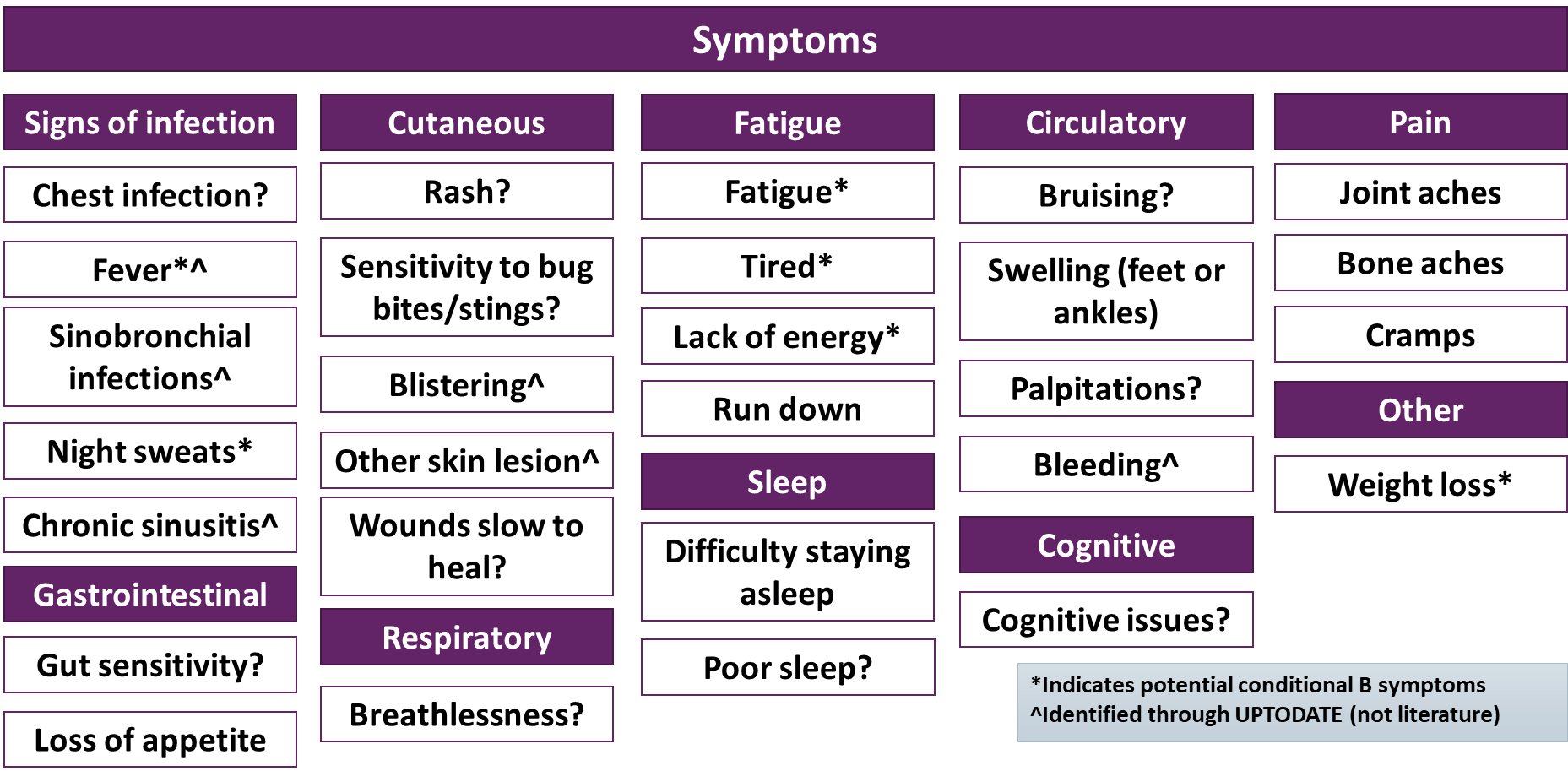


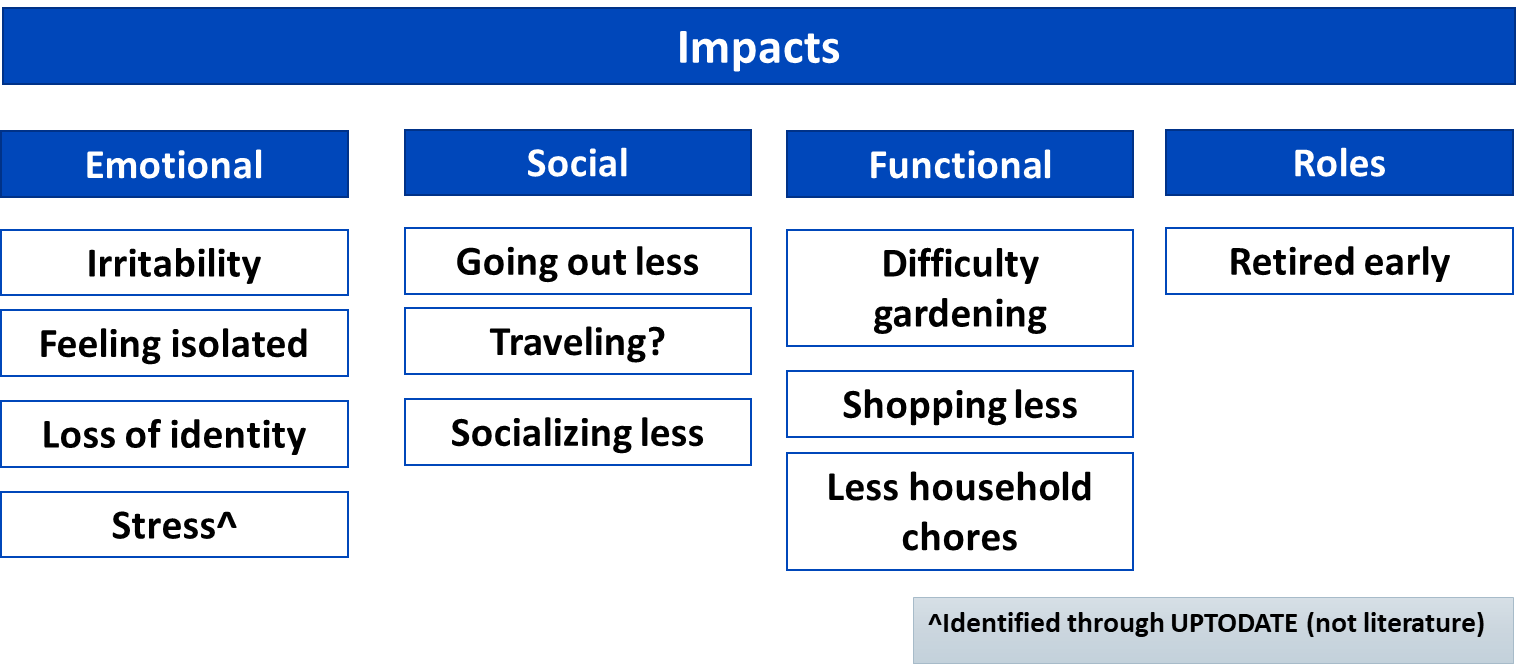


*Figure 1. Draft conceptual models for CLL/SLL*


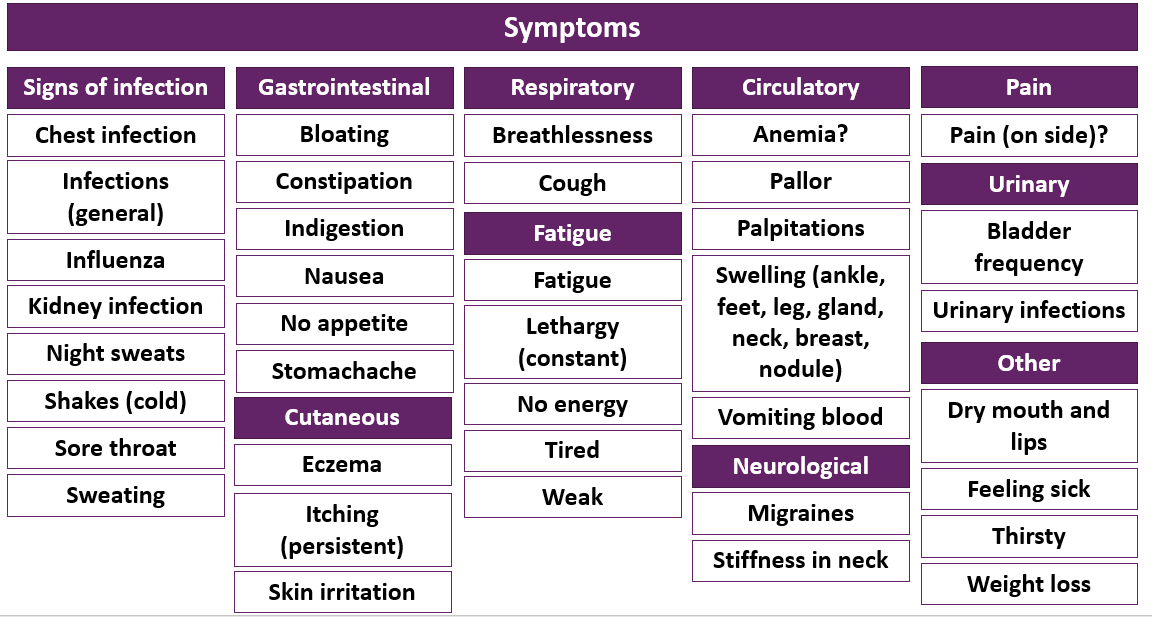


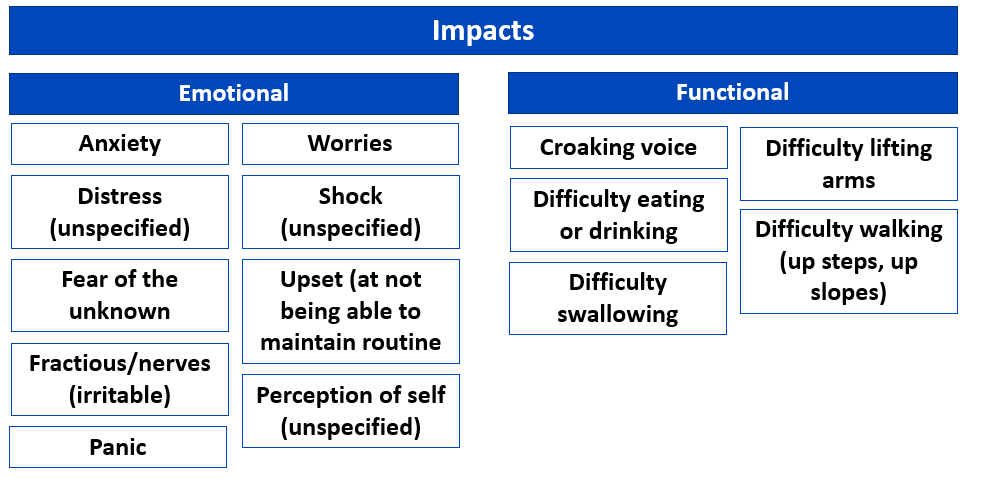


*Figure 2. Draft conceptual models for NHL*
