# Supplementary material for: Mixed-methods research to support the use of new lymphoma-specific patient-reported symptom measures derived from the EORTC item library
Source: J Patient Rep Outcomes. 2024 Jan 22;8:8. doi: 10.1186/s41687-024-00683-2 (PMC10803695; doi:10.1186/s41687-024-00683-2)
Supplement: Supplementary file 6 — Supplementary Material 6: Concept summary tables [file 41687_2024_683_MOESM6_ESM.docx]

S-06 CLL / SLL concept summary tables

CLL and SLL participants reported a variety of symptoms and disease-related impacts. Reported symptoms were grouped under the following sub-domains: swollen lymph nodes, B symptoms, abdominal, pain, fatigue, other anemia-related, bleeding, infections, and other issues. Impact concepts were grouped under the following sub-domains and concepts: Physical function (general physical activities, specific physical activities, general daily activities), Role function (caring, finance, work, other role function daily activities), and Other (psychological, social limitations). Table 1 and Table 2 summarize concepts and related quotations from study participants for CLL/SLL related symptoms and impacts, respectively.

*Table 1. Concepts and quotes for Symptom domain*

| Sub-domain | Concept | Exemplar quote |
| --- | --- | --- |
| Swollen lymph nodes | Swollen lymph nodes | “I had the swollen lymph nodes in my neck, I mean, and they were pretty bad. I just thought it was some kind of an infection, so I didn’t really worry about it. I thought it would just go away and it didn’t.” US105 |
| B symptoms | Chills | “I seem to get those more at night, and it’ll be hot, and then I just suddenly feel chilled, and I know it’s warm in the room, but I just get chilled, so I just throw on another layer.” US131 |
|  | Fever | “I mean, it was a duller type fever. It wasn’t a fever where my – I felt like I was burning up, where I have had that before when I’m fighting an infection. But I would say if you were to take your palm and place it on my forehead, you would definitely feel that I was very warm.” US131 |
|  | Night sweats | “Or sometimes I’ll wake up and I’ll be all sticky and slimy from night perspiration or night sweats.” US110 |
|  | Sweating | “Yeah. It just – sometimes I sweat when it’s cold, so I have to take off a bunch of clothes even though it’s really cold. I’m sweating like crazy.” US105 |
|  | Weight loss | “That all of a sudden I noticed that my clothes were looking looser and feeling looser, and I hadn’t done everything. You know, everybody I know is trying to lose weight, because they ate their way through COVID, and for me it was the opposite.” US131 |
| Abdominal | Gut sensitivity | “Q: Gut sensitivity?  US108: Yes, very much so.” |
|  | Nausea | “Like I said, I would sometimes have the nausea or I would feel kind of sick to my stomach. Wondering – like I wanted to regurgitate, or just nauseous.” US110 |
|  | Upset stomach | “I started getting an upset stomach.” US108 |
|  | Abdominal pain | “Feeling very full even after eating a very small amount, having that pressure feeling and pain. I couldn’t have anything touching my stomach, any waist bands or anything around my middle section, and I’d feel like I needed to lay down, because even sitting up would put added pressure.” US125 |
| Pain | Aching bones and joints | “Q: Any aching bones or joints?  US111: Yes.” |
|  | Joint pain | “But the joint pain is just – it makes it just really hard to move. If I don’t – if I sit in one spot for too long, my joints will really hard for – for me to get – stand up and move, if I stay in one position too long.” US105 |
|  | Muscle cramps | “Q: Any muscle cramps?  US118: Yeah, occasionally.” |
|  | Headaches | “Well, they get bad enough to where I have dim the lights as much as possible and turn volume down as much as possible, so I just kind of shut off a little bit. Usually I have to – it usually goes – it’s usually a couple of hours and I’m – I start feeling better.” US105 |
|  | Sinus pain | “I’m not blowing my nose. It’s just this feeling like – well, I told my husband, it’s like I rubbed hot chili peppers inside my nose or something. They just – I’ve never been aware of my sinuses before, but they’re just kind of there and they’re burning and again, they – all of the symptoms come together, so I wouldn’t have burning sinuses without the other stuff, which is really peculiar.” US107 |
| Fatigue | Decreased energy | “It just kind of limited what I was physically able to do. My stamina and my energy level, I guess I would say, is mostly what was impacted.” US119 |
|  | Extreme exhaustion | “My experience is extreme exhaustion. It’s very hard for me to get anything done, even though I’m a very active – a strong person.” US120 |
|  | Fatigue | “I had the fatigue just slowly get worse and worse. Even to this day, I have the bad fatigue, to the point that I do not want to get out of bed. I do not want to do anything, but I do. I force myself to get up and get dressed and try and do stuff, even though I don’t feel like it. I have absolutely no energy at all. And it’s been that way for about 11, 12 years.” US104 |
|  | Lethargy | “But then it got to a point I couldn’t even imagine the thought of getting in the car and going to the store. That’s when it got so much worse.” US109 |
|  | Tiredness | “The tiredness, the weakness, the having to stay isolated from people so I don’t get anything.” US104 |
|  | Weakness | “It’s usually in my legs. I just feel like if I don’t sit down, I’m going to collapse because my legs feel really weak and like Jell-o.” US105 |
| Other anemia-related issues | Shortness of breath | “But I do get – even walking up a small incline or something, sometimes I’m just like – like I feel like I’m just lightheaded and out of breath as if I’ve just ran a mile. You know, it’s like – so I do get that way sometimes. Or often, actually. Pretty often, actually.” US116 |
|  | Dizziness | “It’s pretty brief, and it’s usually because I’m pushing myself beyond what I should. So it’s a kind of reminder to me to stop.” US107 |
|  | Heart palpitations | “I’ve had some, but not – it hasn’t been ongoing, just every once in a while, I would have heart flutters or something, palpitations.” US117 |
| Bleeding issues | Blood in urine | “…oh, there was blood coming out when I would pee.” US118 |
|  | Bruising | “And every time I did fall, I had bruises the whole length of one leg, knee, and sometimes the second one, and some bruises on the arms. Every time. All bad ones – horrible, bad ones.” US104 |
|  | Brain fog | “…along with the fatigue is a kind of flu-like feeling and a kind of brain fog.” US107 |
|  | Decreased cognitive function | “CLL has taken all that away, and treatment further took it away. I cannot function like that any longer, and my cognitive function from treatment – it’s taken a long time to regain that. I did forget to say that. I’m sorry. That was another effect of treatment, is cognitive and focus, which I have regained mostly, but I’m still in therapy for cognitive. I’m in cognitive therapy because I lost much of it.” US102 |
| Infections | Cellulitis | “In 2014, I had a couple of bouts of cellulitis where it would show up – like I think the first time I noticed it, it was on the inside of my right forearm and it would just be this oval or egg-shaped patch of redness, and the skin would be a little raised there. And it would have like a rough texture. Then it also appeared on my left arm. And I think I was treated with antibiotics for that, and it cleared up. That’s really the only time that that appeared.” US119 |
|  | Feeling sick | “Oh, yes. I had a fever, I had the chills, my body hurt, I just felt very sick. I didn’t feel well. I felt like my body was really having a hard time and unfortunately last July was kind of a peak time of COVID, so everyone immediately thought nothing but COVID.” US121 |
|  | Flu-like feeling | “…along with the fatigue is a kind of flu-like feeling and a kind of brain fog.” US107 |
|  | Increased infections | “I had multiple sinus infections. I had this weird superbug UTI – Klebsiella UTI. I ended up going on IV antibiotics for 10 days.” US101 |
| Other issues | Appetite loss | “I was experiencing lack of appetite, and I’m known at times to eat like a pig. So loss of appetite was extremely concerning” US110 |
|  | Temperature fluctuation/intolerance | “It was never drenching like that, but I would wake up sweating and had the heat and cold intolerances for a while.” US116 |
|  | Rash | “Oh, I have a rash on my arms that’s been there since I got this. For three years I’ve had rashes on both of my arms that do not go away.” US118 |
|  | Weight gain | “Q: Any weight loss?  US120: Weight gain.” |
|  | Sleep problems | “I fall asleep, and then I wake up in the middle of the night, and I’m never quite sure why. But that’s also on and off. A lot of times I sleep a lot. It’s weird. It’s one or the other.” US131 |

Table 2. Concepts and quotes for Impact domain

| Sub-domain1 | Subdomain2 | Concept | Exemplar quote |
| --- | --- | --- | --- |
| Physical function | General physical activities | Exercise | “US130: And if I can get back, I’ll start back with my exercise routine.  Q: The fatigue – does that impact your ability to do your exercise routine?  US130: Yeah, that’s part of it. The severe – yeah, fatigue.” |
|  |  | Physical activities | “Hiking, I couldn’t go as far or as long. I would have to do shorter hikes, and I stuck closer to home. I didn’t do a lot of traveling to do hiking.” US119 |
|  |  | Physical work | “Yeah, I can’t do housework or putting things away. Say some boxes come or delivered or whatever, it sounds funny, but it’s very hard for me to open them and put the thing away, or whatever. So I’m actually hiring people to help me keep my organized and clean. It’s very hard for me to do that kind of stuff. If I clean a bathroom, it wipes me out. So that’s the impact. I can still do mental work, but physical work is very hard.” US115 |
|  | Specific physical activities | Walking | “Walking – I think walking more than three blocks would probably really wear me out. I would consider that strenuous. If I walk a block or two, I think that’d be OK.” US130 |
|  |  | Can’t sit for long | “Just the stomach issues, because it’s just uncomfortable enough that I don’t want to have to sit for long. So I wouldn’t want to be in a car, go for a drive, anything like that” US125 |
|  |  | Don’t want to move | “But when I’m going through a bad day, as I mentioned, where I have extreme fatigue, I’m sitting at the edge of the chair not wanting to move. Just not having the energy to do anything” US110 |
|  |  | Lifting heavy things | “If I had to pick up something more than 50 pounds, I don’t think I could do it, whereas normally I think I could…” US130 |
|  |  | Yard work | “For instance, usually when I would mow the lawn – we have a front and a back yard – I would just bomb through both of them and finish. At the height of my illness, I would have to do one lawn, take a break, or do one lawn one day and the other lawn the other.” US119 |
|  |  | Household work | “…just even household work. I used to have a – OK, every week. There’s like a day. Every Friday, I would come home from work, and I would do the cleaning, and I would do whatever. I just had a routine of everything. Now, it’s more like, OK, well, that will get done on a day that I feel OK. (laughter) So who knows? That might be Friday, or it could be next Tuesday. Who knows when that’s going to happen? We’ll see. It’s a different way of living.” US116 |
|  | General daily activities | Activities limited | “My activities are really, really limited.” US107 |
|  |  | Daily activities | “I still take care of my yard in the summertime. But instead of mowing my yard in a day, it takes me three days. So I’m constantly not finishing things. But I only put it away for a break, and then I go back and do it again.” US108 |
|  |  | Daily routine | “I use to love – it sounds crazy. I used to love shopping or going to even the grocery store or Whole Foods to pick out dinner. Yeah, that would be a chore now, any of that, before COVID. Before treatment, during treatment.” US102 |
| Role function | Caring | Parenting impacted | “I guess, as a parent – for me, personally, I feel like that’s where it’s gotten – I feel like that’s impacted. I don’t think that it’s that noticeable to her as a child because I really try to be like, hey, everything’s fine. La-la-la-la.  But I can feel how tired and stuff. I know that I could do a lot more with her than I do if I didn’t have CLL. So if I wasn’t fatigued, I know that I could be so much more – I’m constantly thinking, oh, yes, if I wasn’t tired, this would be a great thing to do with her. But then I’m like, ugh. So I feel like that’s probably it, that I feel like I can’t be the parent that I want to be because of the fatigue.  I mean, I think that I’m a good parent. I mean, don’t get – I’m not saying – but it’s just I feel like I could be better.” US116 |
|  |  | Taking care of self and others | “Well, in the height of treatment, when I was on both, my mother was gravely ill and dying, and I could not take care of her. So yes. I also was neglectful of my stepson, because I was lucky if I could take care of me. And I did, just not to the extent I normally would. If my husband didn’t cook for me, I probably wouldn’t have eaten, because it would just be that much more energy to expend. But luckily, he’s been great.” US102 |
|  | Finance | Financial impacts | “I also lost income in retirement because of this.  US102 |
|  | Work | Work impacted | “I used to be a corporate officer. I used to be at the C level for a multibillion-dollar publicly traded company. I was a highly functioning executive. I worked seven days a week, and I would work anywhere from 12- to 16-hour days. I’m not bragging – sometimes 18 hours a day. That was my life for years. Jumping on and off planes because I had a global position. I’m not saying this, again, to be bragging or to make it sound glamorous, because it wasn’t. I’m saying it to tell you that was my life. CLL has taken all that away, and treatment further took it away. I cannot function like that any longer, and my cognitive function from treatment – it’s taken a long time to regain that.” US102 |
|  |  | Early retirement | “I had to stop work. I had to retire from my job. I worked at the high school as a special education teaching assistant. And I would have had to retire even outside of COVID, because I had used up like 90 sick days for both the operations and the treatment. So I retired. So that’s been a change in my life.” US101 |
|  | Other RF daily activities | Leisure | “Yeah. I think I’ve just had to switch to more sedentary things and I’m still trying to find some of those things. I’m doing a lot more reading, I guess. Yeah. It’s more of a – just a switch to – instead of more physically active things, more sedentary.” US107 |
|  |  | Hobbies | “Oh, yeah, I would have to say it does. I don’t play golf anymore. I gave up on that. But other than that, I don’t think there’s any other activity I’ve given up on. I may not be able to do it as much or as well, but…” US108 |
| Other | Psychological | Anxiety | “There’s also some mental, you know, issues that go along – that go along. There’s anxiety, there’s stress. You know, I have a little depression. So it impacted me that way. Yes.” US118 |
|  |  | Depression | “Yeah. Everything. It’s just depressing.” US121 |
|  |  | Fear | “Only thing really more that I can add to you is it’s the underlining stress that it causes you of the fear. I’ve never had this diagnosis before. Now I have it. The likelihood of progression and it being part of my life and getting worse is likely, so I live with a lot of stress that – all stress causes symptoms and worry and headaches.” US109 |
|  |  | Lonely | “I’m lonely. I won’t say I’m depressed because I don’t really feel I’m depressed, but I’m lonely. I would like to be around people, but with the COVID I’m afraid to be around people because most of them are not careful enough with who they’re being around. So I could say I’m COVID phobic a little bit. I’m very lonely, but I’m not depressed, that’s probably about it.” US104 |
|  |  | Nervous about driving | “It has made me a little bit nervous about driving sometimes, because I worry about getting lightheaded when I’m driving or – but yeah, it’s not like I feel like I’m going to pass out. I just sort of feel like I need to sit down, stop moving for a while.” US107 |
|  |  | Stress | “I guess I would stress, at least for me, one of the most difficult things is the emotional, and psychological, and financial aspects of it, too.” US117 |
|  |  | Worry | “I’d say I’m more anxious about the future. I’m worried about how much more severe it might get, treatments will stop working…” US131 |
| Other | Social limitations | Ability to interact with others | “In general, yes it has. I don’t want to be around people. I don’t want to get sick. I’m scared if I get sick I’ll catch pneumonia, I’ll die of pneumonia or something terrible. So I haven’t had any interaction. I think COVID plays a big part in that because I’m worried.” US121 |
|  |  | Making plans | “I mean, I was a very, very energetic person. I would travel a lot. I danced a lot. Very social. And now, I can’t plan ahead. So a lot of those things don’t really happen as much anymore. So. Because I can’t make a plan, I have to sort of see, OK, well, I’m going to wake up today and let’s see what happens.” US116 |
|  |  | Social life limited | “On the days when, like I said, I’m not going to want to go for a drive, I’m not going to want to go have dinner with friends or go out to eat or things like that just because I’m hurting.” US125 |
|  |  | Socializing less | “During treatment, that didn’t happen as much, because by nighttime I’d be cooked. I’d be so tired.” US102 |
|  |  | Thinking and communication | “When I’m very stressed out and tired and fatigued, my brain doesn’t think.” US109 |

MCL summary tables

MCL participants reported a variety of symptoms and disease-related impacts. Reported symptoms were grouped under the following sub-domains: swollen lymph nodes, B symptoms, abdominal, pain, fatigue, other anemia-related, bleeding, infections, and other issues. Impact concepts were grouped under the following sub-domains and concepts: Physical function (general physical activities, specific physical activities, general daily activities), Role function (caring, finance, work, other role function daily activities), and Other (psychological, social limitations). Table 1 and Table 4 summarize concepts and related quotations from study participants for MCL related symptoms and impacts, respectively.

*Table 3. Concepts and quotes for symptom domain*

| Sub-domain | Concept | Exemplar quote |
| --- | --- | --- |
| Swollen lymph nodes | Swollen lymph nodes | “And then all of my lymph nodes – my neck, under my arms and my groin – were all swollen. And I can’t really say that they were in pain, but they were so uncomfortable. Just the – it was the pressure of them. It was just so unnerving and uncomfortable.” US124 |
| B symptoms | Chills | “Oh, yeah, the chills – exactly like my COVID symptoms. You get cold, and then my low fever would start. I’d chill. I’d feel like I’m very cold. And then after that, I get my low-grade fever, and I’m warm. So that happened off and on – again, to the level that I knew I had to see what’s going on.” US140 |
|  | Fever | “Sometimes I’d have a fever off and on. Low grade, between 99 and 100. Sometimes 101.” US132 |
|  | Night sweats | “What they call – they tell me I have night sweats where I sweat in the middle of the night or I get a cold and hot and go back and forth.” US123 |
|  | Sweating | “…I was just really tired, and I would have fevers. They went over 101. And sweats…” US126 |
|  | Weight loss | “Oh yes, how it come about is I started losing weight. And I’ve always had not a weight problem, but I’ve always been on the side where you want to always lose weight. All of a sudden just dropping weight and still eating the same was concerning. And I just started having little bouts of fever that was here and there constantly. And it really got to a point I think with the weight loss, my husband was quite terrified and said, there is something wrong with you, and you have to go to the doctor.” US138 |
| Abdominal | Bloating | “Very uncomfortable. You just feel like your stomach’s poofed out constantly. And you feel full, a full feeling.” US133 |
|  | Diarrhea | “There might be times I didn’t feel like eating and felt a little nauseous. Other times, I might have diarrhea, but I couldn’t say that those were symptoms necessarily associated with…” US128 |
|  | Nausea | “It’s like you feel like you want to throw up, but you don’t. You just feel like – maybe like a bad bug, a bad flu, or a bad virus. That’s what it feels like is like you just feel like if you could just get it out. It just makes you feel sick that way, like a virus.” RPV001 |
|  | Abdominal pain | “I had some blood in my stool and some pain in my abdomen…” US124 |
| Pain | Aching bones and joints | “I just feel achy and really kind of – I just feel like my body is breaking.” US138 |
|  | Chest pain | “So I went in to see a primary doctor, because I had a chest pain” US142 |
|  | Muscle cramps | “I get cramps in my legs real bad.” RPV001 |
| Fatigue | Decreased energy | “It was like all of a sudden, I just had no energy.” US137 |
|  | Lethargy | “US128: It felt as though all the energy I had in my body was being consistently drained.  Q: And then, would you use any other words to describe it?  US128: I think draining is the best way, just a flat – 100% complete lack of energy.” |
|  | Mentally exhausted | “And then the hit of, oh, you have an incurable lymphoma here, there’s – there was probably more fatigue in terms of – I don’t know that it was necessarily physical fatigue, but mental fatigue, a lot of it. … mentally it makes me exhausted” US124 |
|  | Tiredness | “I just started to feel very tired for a long time, and I knew something just didn't feel right.” US123 |
|  | Weakness | “No, it was strange. And the symptoms were very minor, I just noticed I was feeling a bit weaker. I could hardly get up and going in the morning and would sit a lot during the day, and I normally have a lot of energy.” US133 |
| Other anemia-related issues | Shortness of breath | “If I try to go up two, three flights of stairs, generally two at a time, I do get a little winded. I do have a little shortness of breath sometimes when I go out walking.” US106 |
| Bleeding issues | Blood in stool | “I had some blood in my stool and some pain in my abdomen, but – and I even told my husband about it. He was like, ooh, you better get that checked out. And I said, yeah. But, honestly, it lasted for like two, three days and was gone. And so I just felt, well, maybe it was something I ate, you know? And it didn’t really come back for well over a month.” US124 |
| Infection | Feeling sick | “Yeah, when it come back, so I was always being monitored with the blood work and stuff to make sure. And then I was also having a little bit of symptoms. When it did come back I was having a little bit of the chills. I felt like I was sick.” US138 |
|  | Increased infections | “Just I get infections. My immune system, I feel it – like right now.” US142 |
|  | Flu-like feeling | “So that was just body aches, fever, runny nose. I just felt like when you have – through all my life, I had flu infections at least once a year for the longest time, since I was a teen.” US142 |
| Other issues | Appetite loss | “There might be times I didn’t feel like eating and felt a little nauseous.” US128 |
|  | Temperature fluctuation | “Q: And then you also mention that your temperature fluctuates. How severe would you say that is?  US123: Again, moderate to severe, I’d feel. It’s just off and on, and you just know your body's fighting and it's just not able to regulate itself just right.” |
|  | Cough | “I had some coughing before. When I had fever and I was coughing, it was all at the same time.” US134 |
|  | Sleep problems | “So it was hard to sleep.” US129 |
|  | Memory problems | “I can’t think of anything else that – my brain doesn’t work like it used to. I can’t say if that’s because I’m getting older or if that is from chemo. I know everybody says chemo brain, and it could be. But in remembering things, I do seem to have like – I can’t think of an example right now, but I do seem to have a little trouble on remembering where did I put this? Which like I said, that could be old age coming on, too. I don’t know. So it’s hard to say if that’s from chemo, from mantle cell, from just getting older.” RPV001 |
|  | Weight gain | “I’d say it probably just kind of happened as soon as all my other symptoms started coming out. So I’d say those kind of coincided with the return of the MCL.” US139 |

Table 4. Concepts and quotes for Impact domain

| Sub-domain1 | Sub-domain2 | Concept | Exemplar quote |
| --- | --- | --- | --- |
| Physical function | General physical activities | Exercise | “I would say exercising, because I like to do that and if I don’t feel good, I don’t do that.” US122 |
|  |  | Physical activities | “it’s essentially lowered my fitness level. I still want to – and I still do – ski and ride my bike and do all that sort of stuff. I just do it a lot slower now.” US129 |
|  | Specific physical activities | Walking | “Like I say, I used to go hiking. I used to walk for 20, 25 minutes. I don’t do that anymore. I’ll probably go walk for at least 10 minutes whenever I feel better.” US142 |
|  |  | Don’t want to move | “It’s days where I just don’t want to do anything really. Just don't want to move.” US123 |
|  |  | Lifting heavy things | “Well, I used to sometimes lift heavy stuff at home, move them around, and those sort of things I avoid right now.” US140 |
|  |  | Yard work | “I’d say probably just like mowing the lawn or anything like cutting hedges and anything that requires any heavy machinery or any type of major work.” US139 |
|  |  | Household work | “I don't cook quite often anymore. I often don’t do a lot of the household responsibilities. My husband does a lot of those things now for us.” US123 |
|  | General daily activities | Activities limited | “Well, I still try to avoid large crowds, I still try to do distancing. I sanitize a lot still, it’s not just because of COVID I'm just trying to avoid getting other things.” US133 |
| Role function | Caring | Taking care of self and others | “US126: No, I can still take care of myself. It’s just a much slower process. It’s just different.  Q: What do you mean by it’s just different?  US126: Well, I don’t have the energy to cook a big meal, let’s say, or the energy to clean up, but I can still dress and do the stuff I need to do. I make my bed every day, and I am able to do things, just not in the capacity I once did.” |
|  | Finance | Financial impacts | “Yes. Yeah, paying for all the CT scans, the PET scans, the doctor visit – yes, it does very much.” RPV001 |
|  | Work | Work impacted | “I think the biggest impact is probably more in my work. Like how much can I accomplish a day? I’ve really had to scale back. I’ve worked reduced hours. I have not.” US129 |
|  | Other RF daily activities | Leisure | Q: Does it affect your participation in leisure activities or hobbies?  A: Maybe a little bit.  Q: And can you just explain – elaborate?  A: Maybe I just tell myself I don't feel like doing it. US141 |
|  |  | Hobbies | “So it interrupts in my planning of getting together, either with family or friends and/or participating in the hobbies that I like to do. Golf. I do play a little bit of basketball with some friends, although at 60, that’s starting to wear off. What used to be serious games is just now friends get – so it’s a get together there.” US128 |
| Other | Psychological impact | Anxiety | “I guess my biggest symptom is worry. With worrying, I guess I can cause anxiety, and with those two things being combined, mentally it makes me exhausted.” US141 |
|  |  | Depression | “Oh, emotional. Yes of course, compared to my old times I am much more depressed and sad. But I try to push myself to be positive and these things. But regarding emotional, yes it affect a lot.” US135 |
|  |  | Discouraged | “Yes, it has. I guess I’m just very discouraged right now. Like I said, I wasn’t in remission for very long, and that just took a big hit on just my whole body. And it took a hit on my family, too, because we thought that, OK, the treatment’s working. I’m in remission. This is great. And then for it to just after five months come back, it’s just very discouraging to me.” US136 |
|  |  | Preoccupied | “I think just my mind if occupied with it. So it’s preoccupied and that’s really about all.” US138 |
|  |  | Sad | “The fatigue makes me feel really sad that I can't do things I could do before.” US103 |
|  |  | Worry | “I guess my biggest symptom is worry. With worrying, I guess I can cause anxiety, and with those two things being combined, mentally it makes me exhausted.” US141 |
| Other | Social limitation | Making plans | “So I can't plan a whole lot ahead of time. That’s probably a very good point. You can't really plan, because you don't know how you're going to feel.” US122 |
|  |  | Social life limited | “Well, not being able to be around my girls’ group. I was part of the Red Hat club, and I, of course, played cards – and just doing things like that and getting out and being around people, and I couldn’t put myself at risk any longer. You know, it’s been four years, and it’s been hard to not participate in that. I still have very close girlfriends that I’m in touch with, but again, I can’t do the things that I once did. And now, with the pandemic, I certainly wouldn’t put myself at that risk.” US126 |
|  |  | Thinking and communication | “That’s a good question. I think that would tie in with sort of the brain fog that I would periodically experience. Some days, I just don’t want to answer emails. I don’t want to have big conversations. I want to just veg.” US129 |
