# Supplementary material for: Mixed-methods research to support the use of new lymphoma-specific patient-reported symptom measures derived from the EORTC item library
Source: J Patient Rep Outcomes. 2024 Jan 22;8:8. doi: 10.1186/s41687-024-00683-2 (PMC10803695; doi:10.1186/s41687-024-00683-2)
Supplement: Supplementary file 7 — Supplementary Material 7: Saturation analysis tables [file 41687_2024_683_MOESM7_ESM.docx]

S-07 CLL/SLL interviews – conceptual saturation analysis

We conducted conceptual saturation on the sub-domain level for symptoms and impacts in five groups of four transcripts. Thirteen concepts emerged in the first group of transcripts, three in the second group, and three in the third group. Conceptual saturation was achieved after interviewing 12 participants. See Table 1 below for details.

Table 1. Conceptual saturation of symptoms and impacts of CLL and SLL

|  | Interviews  1-4 | Interviews  5-8 | Interviews  9-12 | Interviews  13-16 | Interviews  17-20 |
| --- | --- | --- | --- | --- | --- |
| Symptom subdomains | Swollen lymph nodes | B symptoms |  |  |  |
|  | Abdominal | Other issues |  |  |  |
|  | Pain |  |  |  |  |
|  | Fatigue |  |  |  |  |
|  | Other anemia-related |  |  |  |  |
|  | Bleeding issues |  |  |  |  |
|  | Infections |  |  |  |  |
| Impact subdomains | General daily activities | Specific physical activities | General physical activities |  |  |
|  | Finance |  | Caring |  |  |
|  | Work |  |  |  |  |
|  | Other daily role function |  |  |  |  |
|  | Psychological |  |  |  |  |
|  | Social |  |  |  |  |
|  |  |  |  |  |  |
|  |  |  |  |  |  |
| Total new concepts | **13** | **3** | **2** | **0** | **0** |

MCL interviews – conceptual saturation analysis

We conducted conceptual saturation on the sub-domain level for symptoms and impacts in four groups of four transcripts and one group of five transcripts. Almost all the concepts (n=16) emerged in the first group of transcripts, with two emerging in the second group, one in the third, and one in the fourth. Conceptual saturation was achieved after interviewing 16 participants. See Table 2 below for details.

Table 2. Conceptual saturation of symptoms and impacts of MCL

|  | Interviews  1-4 | Interviews  5-8 | Interviews  9-12 | Interviews  13-16 | Interviews  17-21 |
| --- | --- | --- | --- | --- | --- |
| Symptom subdomains | Swollen lymph nodes | Infection |  |  |  |
|  | B symptoms |  |  |  |  |
|  | Abdominal |  |  |  |  |
|  | Pain |  |  |  |  |
|  | Fatigue |  |  |  |  |
|  | Other anemia-related |  |  |  |  |
|  | Bleeding |  |  |  |  |
|  | Other |  |  |  |  |
| Impact subdomains | General physical | Social | Work | Other role function |  |
|  | Specific physical |  |  |  |  |
|  | General daily |  |  |  |  |
|  | Caring |  |  |  |  |
|  | Finance |  |  |  |  |
|  | Psychological |  |  |  |  |
| Total new concepts | **16** | **2** | **1** | **1** | **0** |
