# Supplementary material for: Mixed-methods research to support the use of new lymphoma-specific patient-reported symptom measures derived from the EORTC item library
Source: J Patient Rep Outcomes. 2024 Jan 22;8:8. doi: 10.1186/s41687-024-00683-2 (PMC10803695; doi:10.1186/s41687-024-00683-2)
Supplement: Supplementary file 8 — Supplementary Material 8: Full analysis set description [file 41687_2024_683_MOESM8_ESM.docx]

#### S-08 Demographic characteristics of participants in the full analysis set

Sixty-four participants were recruited in Wave 1 and 2. Due to data collection issues, four participants with CLL/SLL and three with MCL were excluded from the quantitative strand analysis. The full analysis set (FAS) included data from both samples, which included 57 subjects (CLL/SLL population: 27 subjects; MCL population: 30 subjects).

Table 1: Demographics and health characteristics of the CLL/SLL and MCL participants included in the FAS

| Variable | CLL/SLL N=27 | MCL N=30 |
| --- | --- | --- |
| Age |  |  |
| Mean (SD) | 61.19 (7.12) | 63.43 (7.89) |
| Gender identity - n(%) |  |  |
| Female | 18 (66.7%) | 18 (60.0%) |
| Male | 9 (33.3%) | 11 (36.7%) |
| I prefer not to answer | 0 ( 0.0%) | 1 ( 3.3%) |
| Race - n(%) |  |  |
| White/Caucasian | 20 (74.1%) | 23 (76.7%) |
| Black/African American | 3 (11.1%) | 4 (13.3%) |
| Asian | 0 ( 0.0%) | 2 ( 6.7%) |
| American Indian/Alaskan Native | 1 ( 3.7%) | 0 ( 0.0%) |
| Hawaiian Native/Pacific Islander | 1 ( 3.7%) | 0 ( 0.0%) |
| I prefer not to answer | 2 ( 7.4%) | 1 ( 3.3%) |
| Ethnicity - n(%) |  |  |
| Hispanic/Latino | 3 (11.1%) | 7 (23.3%) |
| I prefer not to answer | 2 ( 7.4%) | 0 ( 0.0%) |
| Non-Hispanic/Non-Latino | 22 (81.5%) | 22 (73.3%) |
| Missing | 0 ( 0.0%) | 1 ( 3.3%) |
| Highest education level - n(%) |  |  |
| High school graduate/GED equivalent | 8 (29.6%) | 6 (20.0%) |
| Some college | 4 (14.8%) | 9 (30.0%) |
| Associate degree | 3 (11.1%) | 2 ( 6.7%) |
| Bachelor's degree | 6 (22.2%) | 8 (26.7%) |
| Post graduate degree | 6 (22.2%) | 4 (13.3%) |
| I prefer not to answer | 0 ( 0.0%) | 1 ( 3.3%) |
| Treatment received ^[a]^ - n(%) |  |  |
| Chemotherapy | 14 (51.9%) | 29 (96.7%) |
| Immunotherapy | 9 (11.1%) | 15 ( 0.0%) |
| Chemoimmunotherapy | 2 ( 3.7%) | 3 ( 3.3%) |
| Bone marrow transplant | 0 ( 0.0%) | 1 ( 3.3%) |
| Proteosome inhibits | 1 ( 3.7%) | 0 ( 0.0%) |
| Other | 2 ( 7.4%) | 0 ( 0.0%) |

[a] Participants could choose several treatment options.
