# Supplementary material for: Mixed-methods research to support the use of new lymphoma-specific patient-reported symptom measures derived from the EORTC item library
Source: J Patient Rep Outcomes. 2024 Jan 22;8:8. doi: 10.1186/s41687-024-00683-2 (PMC10803695; doi:10.1186/s41687-024-00683-2)
Supplement: Supplementary file 9 — Supplementary Material 9: Selected quantitative outputs [file 41687_2024_683_MOESM9_ESM.docx]

S-09 Selected quantitative outputs: CLL/SLL and MCL

Figure 1: Adequate distribution of the item responses to the EORTC QLQ-C30 items across the response options despite some items showing a floor effect (FAS, n=57)

Legend: Darker colors indicate higher percentages of participants who endorsed the response.


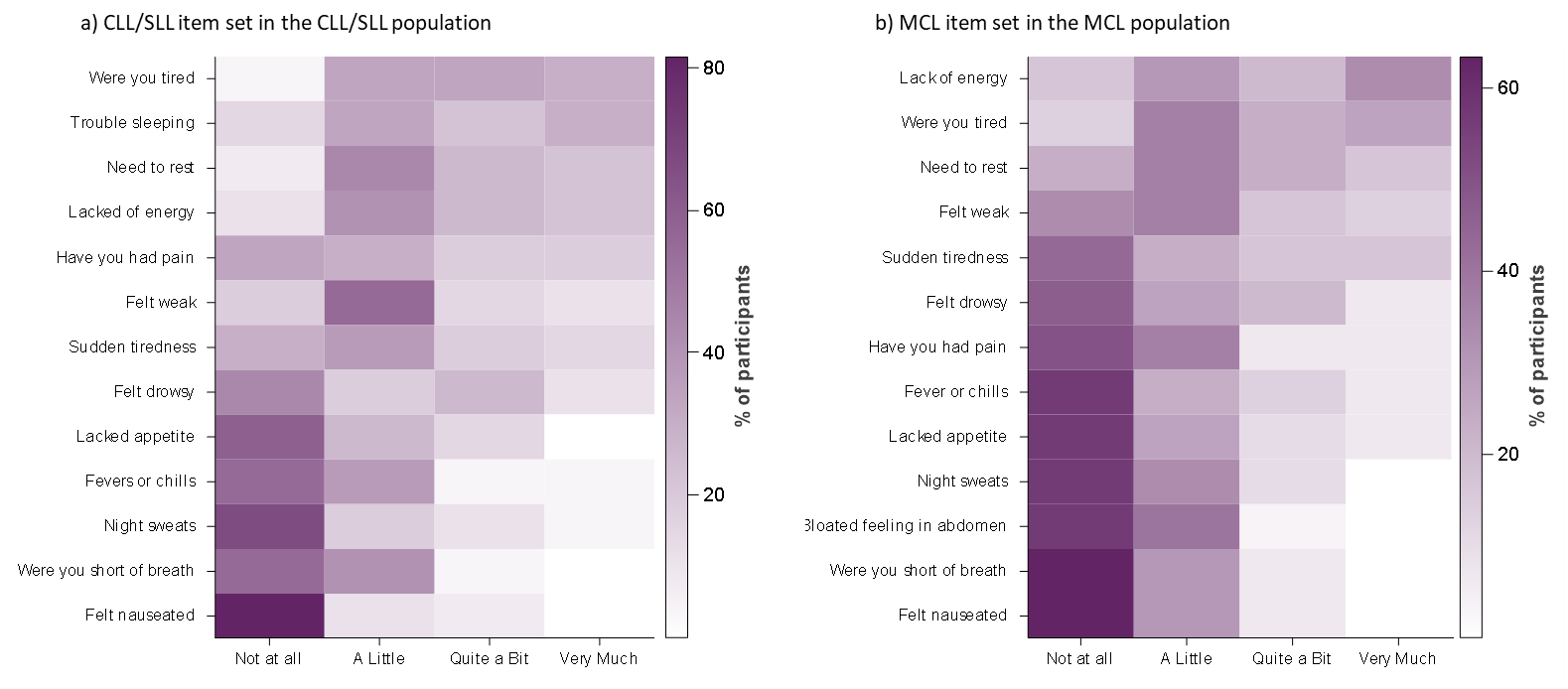


Figure 2: Adequate distribution of the item responses across the response options of a) the CLL/SLL item set in the CLL/SLL participants (n=27), and b) MCL item set in the MCL participants (n=30)

Legend: Darker colors indicate higher percentages of participants who endorsed the response.


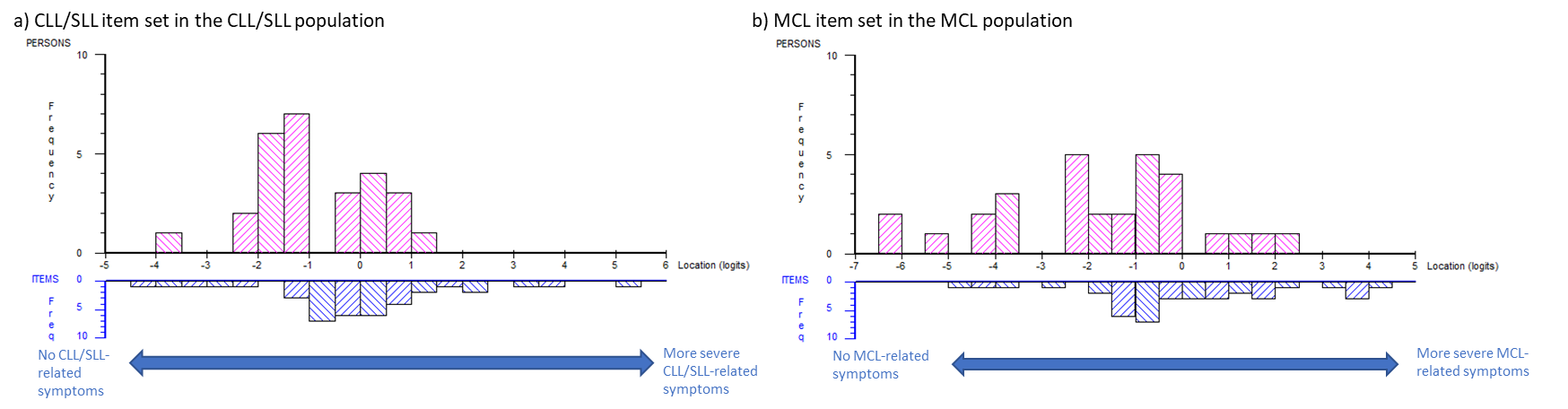


Figure 3: Scale to sample targeting of a) the CLL/SLL item set to the CLL/SLL participant sample (n=27), and b) MCL item set to the MCL participant sample (n=30)

Legend: The upper panel (pink boxes) shows the distribution of the individuals of the study sample over the a) CLL/SLL continuum, b) MCL continuum; The lower panel (blue boxes) shows the respective distribution of the items of the a) CLL/SLL item set on the CLL/SLL continuum, and b) MCL item set on the MCL continuum.


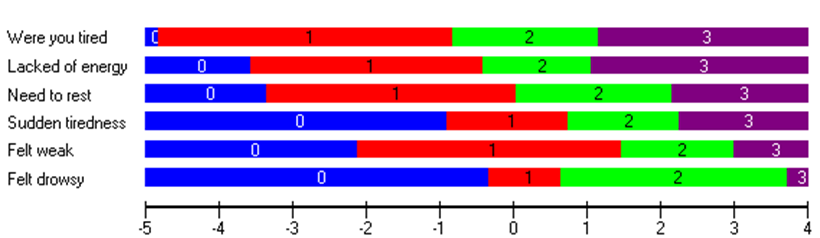


Figure 4: Early outline of fatigue symptoms hierarchy in participants with CLL/SLL and MCL (Fatigue item set; FAS, n=57)

Legend: Darker colors indicate higher percentages of participants who endorsed the response.


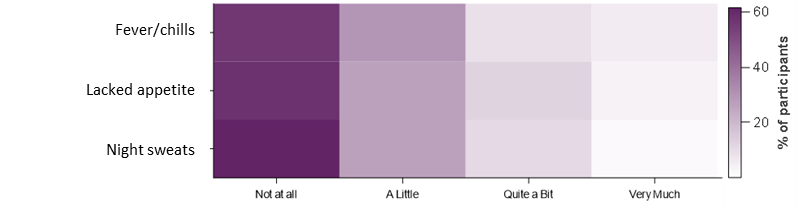


Figure 5: Most CLL/SLL and MCL participants did not experience B symptoms (FAS, n=57)

Legend: Darker colors indicate higher percentages of participants who endorsed the response.
